# Chemical Modification of Microcin J25 Reveals New Insights on the Stereospecific Requirements for Antimicrobial Activity

**DOI:** 10.3390/ijms20205152

**Published:** 2019-10-17

**Authors:** Helena Martin-Gómez, Marta Jorba, Fernando Albericio, Miguel Viñas, Judit Tulla-Puche

**Affiliations:** 1 Institute for Research in Biomedicine, Baldiri Reixac 10, 08028 Barcelona, Spain; marting.helena@gmail.com; 2 Department of Pathology & Experimental Therapeutics, Medical School & IDIBELL Bellvitge, University of Barcelona, Campus Bellvitge, 08907 Hospitalet de Llobregat, Spain; m.jorba.pedrosa@gmail.com; 3 Department of Inorganic and Organic Chemistry–Organic Chemistry Section, University of Barcelona Martí i Franquès 1-11, 08028 Barcelona, Spain; albericio@ub.edu; 4 CIBER-BBN, Networking Centre on Bioengineering, Biomaterials and Nanomedicine, Baldiri Reixac 10, 08028 Barcelona, Spain; 5 School of Chemistry and Physics. University of KwaZulu-Natal, Durban 4001, South Africa; 6 Institut de Biomedicina de la Universitat de Barcelona (IBUB), 08028 Barcelona, Spain

**Keywords:** antimicrobial peptide, microcin J25, epimerization, lasso peptide, mechanism of action

## Abstract

In this study, microcin J25, a potent antimicrobial lasso peptide that acts on Gram-negative bacteria, was subjected to a harsh treatment with a base in order to interrogate its stability and mechanism of action and explore its structure-activity relationship. Despite the high stability reported for this lasso peptide, the chemical treatment led to the detection of a new product. Structural studies revealed that this product retained the lasso topology, but showed no antimicrobial activity due to the epimerization of a key residue for the activity. Further microbiological assays also demonstrated that it showed a high synergistic effect with colistin.

## 1. Introduction

**Figure 1:**
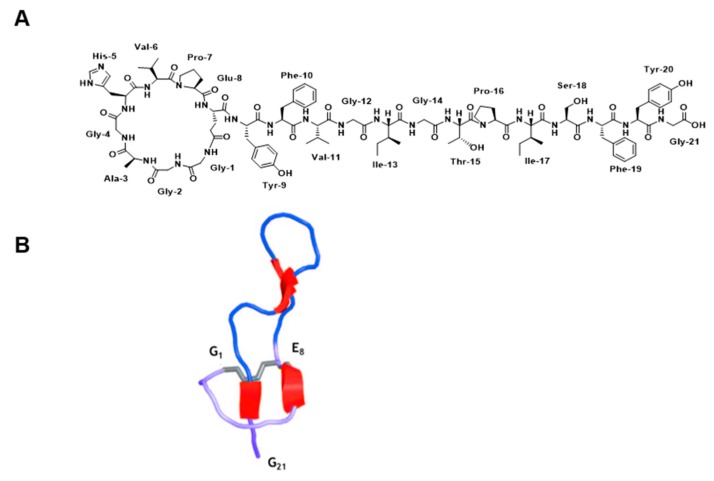
(**A**) Chemical structural and (**B**) ribbon representation of the 3D structure of MccJ25 (Protein Data Bank (PDB) code: 1Q71). The four fragments of antiparallel β-sheets are shown in red. Reprinted (adapted) with permission from Knappe, T.A.; Linne, U.; Zirah, S.; Rebuffat, S.; Xie, X.; Marahiel, M.A. Isolation and structural characterization of capistruin, a lasso peptide predicted from the genome sequence of *Burkholderia thailandensis* E264. J. Am. Chem. Soc. 2008, 130, 11446–11454. Copyright 2019 American Chemical Society [20].

Microcin J25 (MccJ25) is a 21-residue lasso peptide with an 8-residue macrolactam ring, formed between the *N*-terminal Gly1 and the Glu8 side-chain, and a 13-residue *C*-terminal tail, which is threaded through the ring (Figure 1). It is a class II lasso peptide in which the residues Phe19 and Tyr20 are the steric locks. The threaded lasso structure is stabilized by two short double-stranded antiparallel β-sheets. The first comprises residues 6–7 and 19–20, and it is formed between part of the ring and the threaded *C*-terminal tail. The second sheet, which involves residues 10–11 and 15–16, is associated with a β-turn involving residues 11–14, and it forms a hairpin-like structure [1,2,3]. MccJ25 shows antimicrobial activity against a relatively wide range of Gram-negative bacteria. In particular, it exhibits remarkable antibiotic activity towards *Salmonella newport* and several strains of *E. coli*, with minimum inhibitory concentrations typically in the submicromolar range [4].

MccJ25 shows high thermal and proteolytic stability against several proteases, including chymotrypsin, trypsin and pepsin [5]. Moreover, it is stable to highly denaturing conditions, such as 8 M urea and temperatures above 100 °C [6].

Most antimicrobial peptides (AMPs) are characterized by a large number of hydrophobic positive charges and are generally considered to interact with bacterial membrane structures, like indolicidin [7] or LL-37, the active form of human cathelicidin [8]. Once they have crossed the bacterial membrane, they can interfere with internal targets, such as DNA [9,10]. However, MccJ25 has only one positive charge and it inhibits bacterial transcription by interacting with the β′ subunit of the *E. coli* RNA polymerase (RNAP), which is the target of its antibiotic action [6]. MccJ25 also disrupts the inner membrane of *S. newport* by inhibiting several essential processes for cell viability, such as oxygen consumption [11]. In contrast, the peptide inhibits the RNA transcription in many *E. coli* strains [6], without affecting oxygen consumption [12,13]. This peptide has also been reported to exhibit a second mode of action. In this regard, MccJ25 interacts with the membrane, thus depolarizing it and decreasing oxygen consumption. However, the underlying mechanism behind these effects is not yet understood [14]. The uptake of MccJ25 by *E. coli* is dependent on the outer membrane receptor FhuA [15,16,17]. For example, treatment of MccJ25 with thermolysin abolishes its binding to FhuA and results in the suppression of its antibacterial activity, although it is still able to inhibit *E. coli* RNAP activity and *S. newport* respiration *in vitro* [18]. Additional supporting evidence that the Val11 to Pro16 residues are relevant for MccJ25 uptake emerged from an extensive mutational scanning analysis [19].

To gain further insight into the stability and mode of action of the lasso peptide MccJ25, we performed a chemical modification by treatment with basic conditions (0.5 M NaOH), which produced an alteration in the topology of the peptide and the consequent modification of biological activity. We examined how the alteration in the structure affected the mechanism of action through its comparison with the natural lasso peptide.

## 2. Results

### 2.1. Chemical Treatment

The RP-HPLC analysis denotes that the structure of the native lasso peptide was affected by the harsh extended treatment with a base (Figure S1). In this way, a new peak was detected (Figure S2A,B). After being left overnight, the ratio between the starting material and the new product was 1:1 and even with the addition of more NaOH or an increase in reaction time, no reaction progress was observed. The new product, which is shown in Figure S2A, was purified and isolated. RP-HPLC revealed that the new compound and the native peptide did not co-elute, and distinct chemical equivalence was confirmed by different retention times (t_R_), even though these two compounds had the same molecular mass (2107.8 Da) (Figure S2C). Furthermore, the new compound was subjected to the same treatment with basic conditions, and no differences were observed after overnight. This result suggested that the modification was not reversible.

### 2.2. Minimum Inhibitory Concentration (MIC)

The antimicrobial activity was then evaluated against eight Gram-negative strains, among them four were Multidrug-Resistant bacteria (MDR). As expected, the native MccJ25 displayed activity against four of them: two *E. coli* and two *S. enterica* strains (Table 1). On the contrary, the new compound showed the lowest activity against the eight strains tested. Its MIC values were in all cases higher than 128 μg/mL. Thus, we addressed whether the loss of activity was due to the inability of the new compound to penetrate the target bacteria, whether it was caused by changes in its antibacterial properties exerted inside the bacterial cell, or if it was a combination of both factors.

### 2.3. Synergy Study

Colistin, a cationic antimicrobial peptide, even at low concentrations severely disrupts the outer membrane, allowing chemicals to penetrate Gram-negative bacteria. The inability to penetrate bacteria is a major cause of resistance. Although the medical use of colistin was abandoned 30 years ago as a result of its very high toxicity, it was rescued when superbugs (extreme pan-resistant strains) emerged. The recovery of colistin has spurred research into various strategies that allow the preservation of its antimicrobial action and a reduction of its negative side effects. One such strategy involves its combination with drugs with low toxicity to achieve synergistic activity. Colistin may serve as a “door opener” even at concentrations at which the negative side effects are negligible. A checkerboard assay using a combination of the new compound and colistin was performed against the *E. coli* MDR 39255 strain and the *S. enterica* ATCC 13076 (American Type Culture Collection) strain, which are susceptible to native MccJ25 but not to the new compound, as shown in the MIC assay (Table 1). Moreover, two strains resistant to native MccJ25 (*E. coli* MDR 208691 and 239910) were also tested in order to compare results in both types of bacteria.

The fractional inhibitory concentration (FICi) values for the bacterial strains tested with combinations of the new compound and colistin are shown in Table S1. According to the international standard, FICi values below 0.5 should be interpreted as synergistic (see “Methods” section for more details) [21]. Thus, these experiments indicated a synergistic effect between colistin and the new compound.

These results show that the new compound retained the ability to inhibit the growth of these two strains (*E. coli* MDR 39255 and *S. enterica* ATCC 13076), although it was unlikely to have the capacity to enter the bacteria through the outer membrane, the reasons for that have to be investigated and may probably involve its ability to bind the receptor FhuA. In contrast, when the new compound was combined with colistin, it penetrated the outer membrane, thereby demonstrating that the antibacterial mechanisms of action were conserved after the chemical treatment. On the other hand, when the combination of colistin and the new compound was tested in bacteria resistant to native Mcc25, namely *E. coli* MDR 208691 and *E. coli* MDR 239910, a weak synergistic effect was observed. These results could be explained by the change in the peptide topology observed by RP-HPLC.

### 2.4. Minimal Biofilm Eradication Concentration (MBEC)

The MBEC values were, in all cases, greater than 128 μg/mL. Such value is thousands of times greater than the inhibitory values against planktonic bacteria. Therefore, it should be assumed that neither MccJ25 nor the new compound can be considered antibiofilm agents.

### 2.5. Stability Assays

In order to understand the loss in biological activity of the new compound, the stability and structure of the peptide was analyzed. Initially, the chemical treatment was believed to have converted the lasso peptide into a branched cyclic peptide. However, the carboxypeptidase assay rendered the same cleavage pattern for the native peptide and the new product, corresponding to the loss of 81 Da (Figure S3). This loss was attributed to the double cleavage in the loop between the amide bond of Phe10-Val11 and Val11-Gly12, resulting in the loss of Val11 and maintenance of the threaded structure. We obtained a total conversion carboxypeptidase Y treatment, in contrast to previous studies in which only minor traces of the (MccJ25–18) Da were detected [22]. Although the two peptides showed the same cleavage pattern, their t_R_ differed, as shown in the co-injection in Figure S3C. Both peptides were also thermally stable for 4 h at 95 °C (Figure S4). Carboxypeptidase Y treatment was also performed on the resulting peptides after heating for 4 h at 95 °C. In this regard, the same cleavage pattern as in Figure S3 was observed.

**Figure 2:**
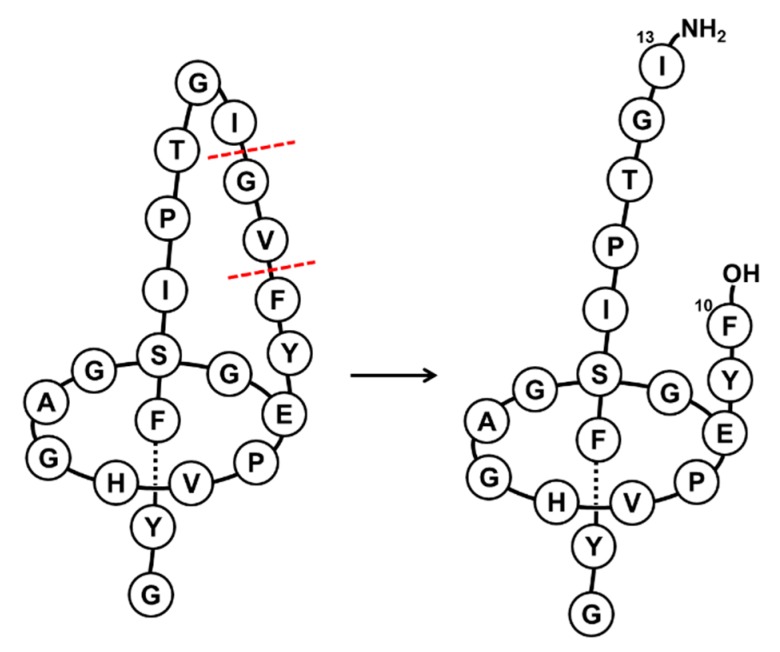
Schematic representation of thermolysin cleavage in the native MccJ25 and in the new compound. Dashed red lines indicate the cleavage sites.

To further characterize the new compound, both this peptide and the native MccJ25 were subjected to proteolytic hydrolysis with stronger proteases, such as pepsin and thermolysin. As expected from the pepsin treatment, no hydrolysis was observed, even after several hours at pH 2 at 37 °C [5]. Given this result, the temperature was increased to 50 °C, as pepsin is stable at this temperature. However, no cleavage was detected after 19 h, thereby indicating that both the native peptide and new compound were stable (Figure S5). These results ruled out the formation of the unthreaded structure. On the other hand, thermolysin treatment produced the same cleavage pattern for the two peptides, corresponding to the loss of 137 Da (m/z 1969.9) (Figure S6A,B) [23,24,25]. This loss could be attributed to the double cleavage between Phe10-Val11 and Gly12-Ile13, with the loss of Val11-Gly12 and the subsequent gain of 18 Da. This cleavage led to a structure with two fragments stabilized by the steric hindrance of Phe19 and Tyr20 and the presence of non-covalent interactions (Figure 2) [24]. Of note, after the treatment with thermolysin, the two peptides showed the same t_R_ value (Figure S6C). This result indicated that the cleavage led to the same structure, thereby suggesting that the variable part of the compound was removed during hydrolysis (Val11-Gly12). In this regard, this result can be explained only by the inversion of the chiral center of Val11. This question will be addressed in future structural studies.

### 2.6. MS/MS Analysis

The tandem mass spectrometry (MS^2^) fragmentation spectra revealed that the main fragmentations took place around Pro16 (Figure S7). The same fragmentations were obtained for the two peptides, but with different intensities (Table S2). These fragmentations were characteristic of a lasso structure, since they contained both the *N*-terminal and *C*-terminal parts of the molecule. The steric hindrance between the ring and the bulky side chains of a putatively threaded *C*-terminal part of the peptide would explain this remarkable fragmentation pattern. Regarding the native MccJ25, the fragmentations obtained were in agreement with those already described in the literature [1,24].

The MS^3^ spectra of the main fragmentations showed the same pattern for the native MccJ25 and the new product, the only difference being that the MS^3^ fragmentations of b_13_ + y_6_ were more intense for the former (Figure S8). On the basis of these results, we confirmed that the new compound showed the lasso topology and that, with the exception of its t_R_, its structure was very similar or even the same as that of the native MccJ25.

### 2.7. Ion-Mobility Mass Spectrometry (IM-MS) Analysis

Small changes in collision cross section (CCS) values were observed upon increasing the charge state of both the native MccJ25 and the new peptide (Table S3). This feature was demonstrated by the low range of CCS values (∆Ω) and the concomitant low ∆Ω/Ω value (Table S4). Small changes in CCS values are characteristic of lasso peptides, as an increase in charge state brings about more unfolding and, consequently, lower drift times (t_d_). However, due to the organized and constrained structure of lasso peptides, the increase in the CCS was very low compared to branched cyclic or lineal peptides. The ∆Ω/Ω value of the new compound was within the accepted range for lasso peptides (0 to 10.9%) [26]. It was therefore deduced that this peptide had a lasso structure.

**Figure 3:**
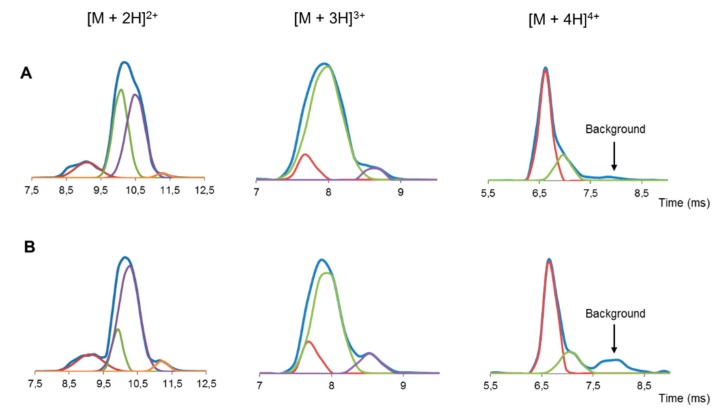
Drift times profiles (blue trace) and fitted peaks (red, green, purple and orange trace) of double, triple and quadruple protonated ion of (**A**) the new compound obtained after treatment with a base and (**B**) native MccJ25. Background is the residual drift time profile, in which no more peaks could be fitted.

The number of multiple conformations was higher for lower charge states, as expected due to the larger number of available protonation sites and the coexistence of several protomers [27]. However, when the charge state increases, narrow ion mobility peaks, associated with the existence of few conformations, are expected (Figure 3).

IM-MS revealed that the new compound showed the same features as those of native MccJ25, thus allowing us to confirm once again the lasso structure of the former.

### 2.8. Circular Dichroism (CD) Spectroscopy

**Figure 4:**
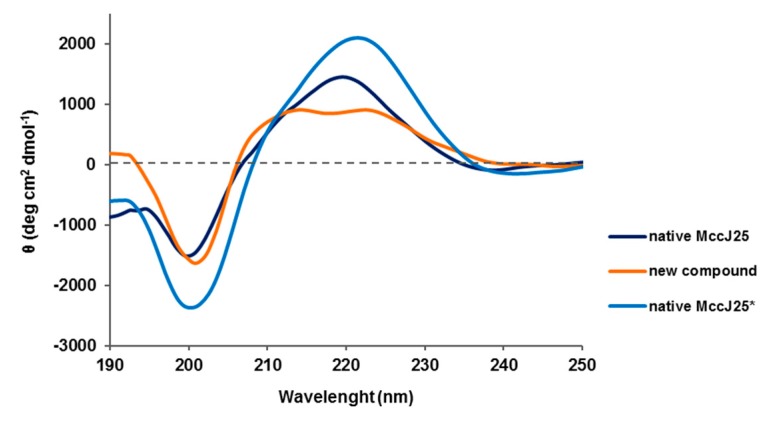
CD spectra of new compound after treatment with a base and native MccJ25 in methanol. * in H_2_O with 5% TFE.

The CD spectra of the two peptides were recorded in methanol [28,29]. They displayed a clear minimum at 200 nm, which is characteristic of unstructured peptides, indicating a predominant random coil state (Figure 4). The native MccJ25 in methanol showed a maximum at 220 nm and a smooth positive shoulder at 210 nm. These bands have been previously attributed to the Phe L_α_ and Tyr L_α_ transitions [30]. However, for the native MccJ25 in H_2_O with 5% of 2,2,2-trifluoroethanol (TFE), the maximum was located at 222 nm and was more intense than in methanol. In addition, the shoulder at 210 nm was not marked. On the contrary, the positive band at 220 nm for the new compound was displaced to 225 nm and the one at 210 nm was more defined and clearer than for the native peptide. This behavior suggested that the L_α_ transition of these residues had greater relevance for the new compound than for the native MccJ25.

In conclusion, the CD spectra revealed some differences in the positive bands of the two peptides, but the clear minimum at 200 nm indicated a random coil structure for both. The changes in the positive bands may indicate distinct topologies.

### 2.9. Nuclear Magnetic Ressonance (NMR) Spectroscopy

The NMR data for the structural characterization of the new compound were recorded in methanol-d_3_ and compared with those of the native MccJ25 (Tables S5–S8 and Figures S9–S14) [1,24,25,28,31]. The resonance assignments of the native MccJ25 were identical to those of the structure already published (PDB code 1PP5) [31]. Given our previous results from the thermolysin assay, we hypothesized that the difference between the two peptides may reside in the peptide loop, between residues 10–12 in particular, which are involved in the β-hairpin, and that after thermolysin treatment the cleaved product showed the same t_R_.

**Figure 5:**
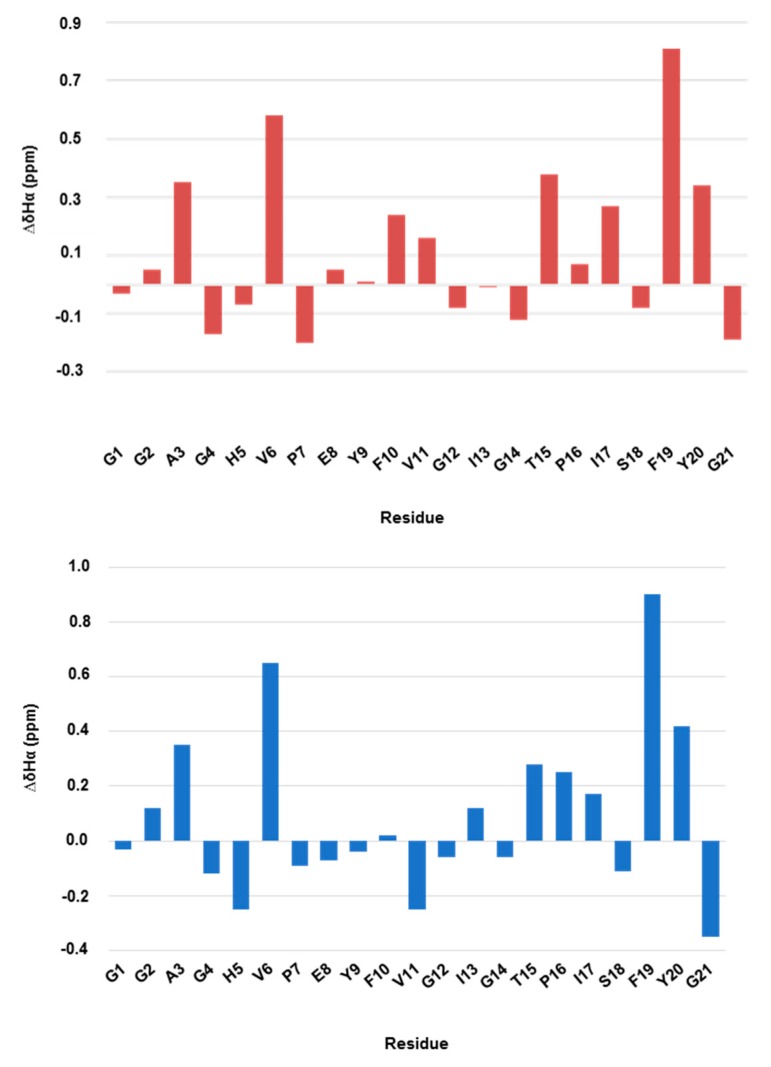
Histograms of chemical shift deviations from random coil for ∆Hα in the native MccJ25 (red) and new compound (blue) in methanol. Random coil values were obtained from Whishart et al. [32].

Regarding ^1^H assignment, the chemical shift deviation values of αH of the loop residues in the new compound differed to those of the native structure (Figure 5). The most remarkable residue was Val11, indicating that the β-hairpin had been lost, as demonstrated by the negative value [18]. On the other hand, the structure of the ring and the tail had been conserved, including the β-hairpin between residues 6–7 and 19–20, as shown by the high positive values in Figure 5. In addition, the overlapping of several peaks was observed for the new compound in the amide proton region around 7.80, 7.90 and 8.40 ppm (Table 2).

Evidence that the tail was threaded through the ring in the new compound was provided by the presence of the characteristic Nuclear Overhauser Effect (NOE) cross-peaks between residues 1–8 and residues 19–21 (Figures S15–S17A) [24]. For example, the NOEs between NH Tyr20 and αH Gly2 and δH Phe19 and βH Ser18 were also more intense than for the native MccJ25. Moreover, some NOEs, such as βH Phe19–NH Glu8 and δH Tyr9–αH Gly14, were detected only in the new compound (Figure S17B). Analysis of the two β-hairpins in the native MccJ25 structure reveals clear differences (Table S9). The disappearance of the β-sheet in the loop can also be detected by the lack of NOEs between αH Val11 and δH Pro16 (Figure S17C). The two Pro (7 and 16) in both structures adopted a trans conformations, as shown by the presence of the NOE between the δH Pro(i) and the αH residue(i-1).

With respect to Val11, various NOE cross-peaks were observed, thereby revealing a distinct topology for the new compound (Table S10). In general, the intensities of the NOEs for the new compound were lower than those of the native one. However, the new compound showed a stronger connectivity with Phe10 and Thr15 (Table S11). The ring protons of Phe10 adopted a different conformation. In the new compound, this residue interacted with δH Pro16, βH Ile17, NH Val11 and αH Ala3, while in the native peptide it interacted with αH, γH Pro16, βH, γH Glu8, βH Phe19 and NH Thr15 (Figure S17D).

All the results pointed to the epimerization of Val11 during the treatment with a base, a process that yielded a new peptide topology. This epimerization explains the loss of the β-sheet in the loop region, producing a lack of organization and consequently a high signal overlap.

### 2.10. Structure Calculation

**Figure 6:**
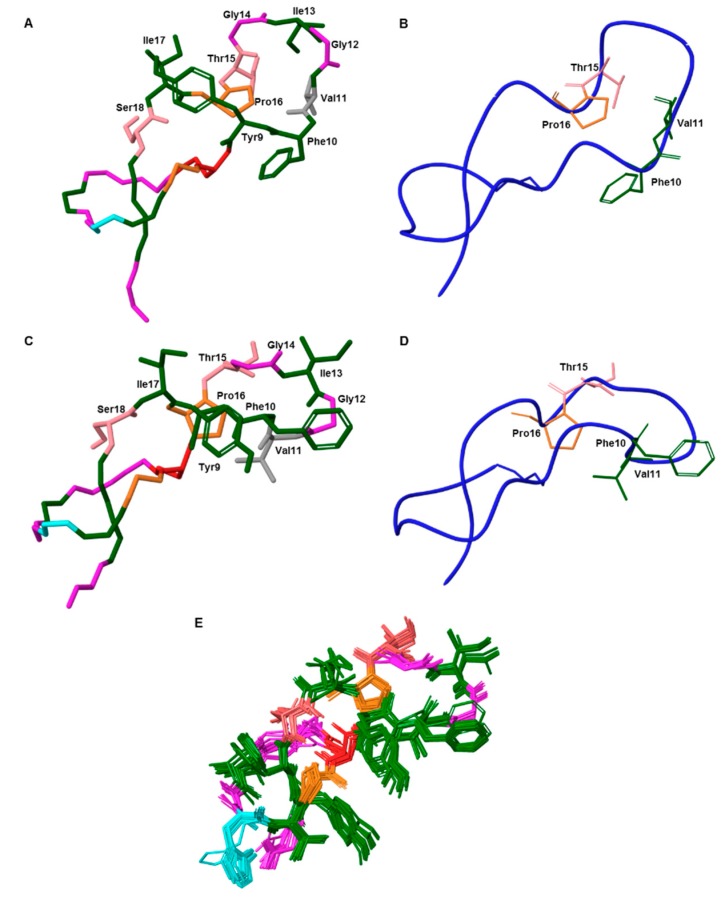
(**A**) and (**C**) 3D structure of the native MccJ25 (PDB code 1PP5) and NMR-derived lowest-energy structure of the new compound, respectively. Only the side chains of the loop are shown. (**B**) and (**D**) Ribbon representation of the native peptide and new compound, respectively. The four residues involved in the β-sheet located in the loop are shown. (**E**) Ensemble of the 20-lowest energy structures derived from the NMR restraints of the new compound. The maximum RMSD value between structures 1–20 is 0.6 Å. Aromatic and hydrophobic residues are shown in green, negatively charged residue (Glu8) in red, positively charged residues (His5) in blue, polar residues in light pink and non-polar residues in pink (Gly) and (Pro). In (**A**) and (**C**) Val11 residue is shown in grey.

The structure of the new compound was determined using a macrocyclic conformational sampling tool [33], and the constraints are described in Table S12. The lowest-energy structure was compared with the native MccJ25. The lasso structure was maintained and the modification was mainly in the loop region (Figure 6). The interaction between the side-chains of Val11 and Pro16, characteristic of a β-sheet, was not present. The αC of Val11 underwent racemization and the side-chain of Val11 adopted a new orientation. Furthermore, the high root-mean-square deviation value (RMSD = 8.5 Å) exhibited that the structures of the native peptide and new product differed.

The Ramachandran plots for the two peptides differed (Figure S17). The native MccJ25 showed a well-defined and compact structure, with the individual backbone conformations of all residues located mainly in highly favored regions. On the contrary, the new compound showed more dispersion. The β-sheet in the loop was absent, and Pro16 was observed to be involved in a right-handed α-helix and Val11 in a left-handed one (Figure S18). Gly12 and Ile17 also adopted a left-handed α-helix conformation. On the other hand, the ring residues 5–8 of the new compound remained in the same conformation as that of the native structure.

## 3. Discussion

We have demonstrated that basic chemical treatment of MccJ25 produced a new compound that maintained the threaded lasso structure but lost its antimicrobial activity. Three independent properties corroborated the presence of the lasso topology: high proteolytic stability; identical MS^2^ fragmentation pattern; and ion-mobility spectra as the native MccJ25. Circular dichroism revealed slight differences in the positive bands between the two peptides, thereby pointing to distinct topologies.

The microbiological results showed that the new compound did not exert antimicrobial activity, as shown by its incapacity to penetrate the outer bacterial membrane. However, when used in combination with colistin, the new compound did show antimicrobial activity. These findings were consistent with the NMR results, in which a change in the loop topology, caused by epimerization at the Val11 residue, was detected. This observation suggests that Val11 is highly base-sensitive and is essential for conferring antimicrobial activity, because it governs binding to the FhuA receptor [34]. This epimerization produced a conformational change on the loop between Val11 and Pro16, which is in agreement with previous evidence that this section is relevant for the antimicrobial activity of MccJ25 [19]. Ile13 is also an essential residue for the uptake of MccJ25 into cells [35]. The new compound showed an alteration in the orientation of the Ile13 side chain, which led to the suppression of biological activity.

The weak synergistic effect observed when the new compound was used in combination with colistin and assayed on resistant bacteria (*E. coli* MDR 208691 and *E. coli* MDR 239910), is consistent with the hypothesis that penetration is the main handicap for antimicrobial activity. In the case of such resistant strains the entry is not the reason for resistance. Although the microbiological results are preliminary, we propose that the combination of bacteriocins with “door openers”, such as colistin, may provide a promising strategy to fight multidrug-resistant infections. In this regard, the treatment of such infections is becoming increasingly unsuccessful and has thus emerged as a key challenge in current microbiological research [36].

## 4. Materials and Methods

### 4.1. Heterologous Expression of MccJ25

Overnight cultures were prepared in Luria broth (LB) medium containing 17 µg/mL of Chloramphenicol at 37 °C through inoculation with *E. coli* BL21 cells carrying the MccJ25 plasmid (pTUC202). Subsequently, 500 mL of M9 minimal medium ((17.1 g/L Na_2_HPO_4 ×_ 12 H_2_O, 3 g/L KH_2_PO_4_, 0.5 g/L NaCl, 1.0 g/L NH_4_Cl, 1.0 mL/L MgSO_4_ 2 M, 0.2 mL/L CaCl_2_ 0.5 M at pH 7.0), after autoclaving 10 mL/L glucose solution (40% *w/v*), 0.2 mL/L vitamin mix (Table S13), and 17 µg/mL amphenicol) were inoculated with 5 mL of culture and incubated for 3 days at 37 °C. Once the OD_600_ reached ~1.5, cells were harvested by centrifugation (2 × 20 min at 7000 rpm), and the supernatant was treated with 10 mL of XAD-16. After 1 h of shaking at 100 rpm, the supernatant was removed by filtration, and the resin was washed with water and extracted with 50 mL of MeOH. Solvent was removed under vacuo. Dried extract was resuspended in a total of 1 mL of MeOH-H_2_O (1:1, *v/v*), cleared by centrifugation, and analyzed by RP-HPLC. For the isolation and purification of MccJ25, the expression was carried out with 6 L of M9 minimal medium. In this case, the procedure was the same, and the dried extract was resuspended in 6 mL of MeOH-H_2_O (1:1, *v/v*), cleared by centrifugation and filtration, and then subjected to preparative HPLC.

### 4.2. Minimum Inhibitory Concentration (MIC)

MIC values were determined by the broth microdilution method and interpreted following the guidelines of the Clinical & Laboratory Standards Institute (CLSI) and European Committee on Antimicrobial Susceptibility Testing (EUCAST) [37]. The different strains were grown in Mueller-Hinton broth (MHB) overnight at 37 °C with shaking at 200 rpm. The bacterial cultures were then adjusted to OD_625nm_ of 0.08–0.1 and diluted 1:100 in fresh MHB medium. Next, 5 µL of each diluted suspension was added to 96-well plates previously filled with MHB and serially diluted peptides. The plates were incubated at 37 °C for 24 h, after which the MIC was determined macroscopically, based on the visually turbidity of the wells. All experiments were performed in triplicate.

### 4.3. Synergy Study

A checkerboard test was used to determine the fractional inhibitory concentrations (FICs) of colistin in combination with the new compound. Each well in a 96-well plate was inoculated with 100 µL of a bacterial inoculum of 1 × 10^5^ CFU/mL, and the plates were incubated at 37 °C for 24 h. The FIC was calculated after identifying the first well in each row without growth (MIC), following Equations (1) and (2).

(1) $$FIC_{A}=\frac{MIC\;drug\;A\;in\;combination\;}{MIC\;drug\;A}$$

(2) $$FIC_{B}=\frac{MIC\;drug\;B\;in\;combination\;}{MIC\;drug\;B}$$

The FIC index (FICi) values were calculated by adding the FIC A (collsitin) to the FIC B (peptide). FICi values were interpreted as follows: FICi < 0.5, synergistic; FICi ≥ 0.5 and < 4, no interaction; FICi > 4, antagonistic [38]. This assay was performed in triplicate.

### 4.4. Minimal Biofilm Eradication Concentration (MBEC)

MBEC was determined as described by Moskowitz et al. [39]. Bacterial biofilms were formed by immersing the pegs of a modified polystyrene microtiter lid into 96-well microtiter plates, each containing 200 µL of Muller-Hinton broth cation adjusted (MHBCA), followed by incubation at 37 °C for 24 h. Pegs were then gently rinsed in a 0.9% *w/v* NaCl solution and biofilms were exposed to a range of concentrations of antimicrobials for 24 h at 37 °C. Pegs were again rinsed with a 0.9% *w/v* NaCl solution and biofilms were removed by 10 min sonication. Recovered bacteria were incubated for 24 h at 37 °C. Optical densities at 620 nm were measured in order to determine MBEC values, defined as the lowest concentration of antimicrobial that prevented bacterial regrowth from the treated biofilm. All experiments were performed in triplicate.

### 4.5. NMR Spectroscopy

1D and 2D NMR spectra were acquired on a Bruker 600 Avance II Ultrashield, equipped with a cryoprobe. Chemical shifts (δ) are shown in parts per million (ppm) using tetramethylsilane (TMS) as internal standard.

1.5 mg and 1.0 mg of the native MccJ25 and the new compound, respectively, were dissolved in 600 µL of methanol-d_3_ to a final concentration of 1.2 mM and 0.86 mM, respectively. The spectrum was recorded at 25 °C and the residue assignments were obtained from 2D total correlated spectroscopy (TOCSY), while 2D nuclear Overhauser effect spectroscopy (NOESY) permitted sequence-specific assignments. ^13^C resonances were assigned from 2D ^13^C–^1^H HSQC spectra. The TOCSY and NOESY mixing times were 70 and 250 ms, respectively.

### 4.6. Ion-Mobility Mass Spectrometry (IM-MS)

IM-MS experiments were recorded on a Synapt G1-HDMS mass spectrometer (Waters). 0.1 mg of peptide was dissolved in 500 μL of water at 100 μM. Samples were then diluted 1/10 in water -acetonitrile (H_2_O-ACN) (1:1) with 4% of formic acid and adding 200 mM and 400 mM sulfolane up to 10 μM. Samples were directly injected into the instrument using a Triversa Nanomate system (AdvionBioSciences, Ithaca, NY, USA) as the interface. Ionization was recorded in positive mode using a gas pressure and spray voltage of 0.5 psi and 1.75 kV, respectively. Source temperature, extraction cone and cone voltage were set to 20 °C, 3 V and 40 V, respectively. Transfer and trap collision energies were set to 4 V and 6 V, respectively. Ion-mobility spectrometry (IMS) and trap gas flows were 25 and 8 mL/sec, respectively. The pressure in the Trap and Transfer T-Wave regions were 5.97× 10^−2^ mbar of argon and the pressure in the IMS T-Wave was 0.478 mbar of N_2_. The wave amplitude was a linear ramp from 1.0 to 28.0 V. The traveling wave operated at a velocity of 250 m/sec. The bias voltage for getting in the T-wave cell was 15 V. Cesium iodide was used to calibrate the instrument over the *m/z* range of 200–3000 Da. MassLynx 4.1 SCN 704 (Waters, Spain) and Drift scope version 2.4 software were used for data processing.

### 4.7. MS/MS Analysis

MS^2^ and MS^3^ analyses were carried out on a linear trap quadrupol fourier transform(LTQ-FT) Ultra mass spectrometer (Thermo Scientific). 0.1 mg of peptide was dissolved in 500 μL of water at 100 μM. Then, 10 μL was diluted 1/1 with ACN (1% formic acid) to obtain a concentration of 50 μM (20 μL). Solutions were directly injected into the instrument using a Nanomate system (AdvionBioSciences, Ithaca, NY, USA) as the interface. Ionization was recorded in positive mode using a gas pressure and spray voltage of 0.5 psi and 1.75 kV, respectively. Voltage and capillary temperature were set to 44 V and 200 °C, respectively. Collision-induced dissociation (CID) was used as a fragmentation technique. Data were recorded using Xcalibur software vs.2.0SR2 (ThermoScientific).

### 4.8. Structure Calculation

The structure was calculated with the Schrödinger Suite Macrocyclic Conformational Sampling tool [33]. The coordinates were extracted from the native MccJ25 solution NMR structure (PDB ID: 1PP5). The stereochemistry of Val11 was manually mutated to R. The resulting structure was used as input for structural modeling. NOEs were classified as strong, medium and weak (upper limits for structure calculation were set as 2.2 Å, 3.0 Å and 4.0 Å, respectively), and were then applied to the structure (Table S12). Optimized potentials for liquid simulations (OPLS) 2005 force field and Generalized Born surface area (GBSA) for electrostatic treatment were used to generate a total of 56 conformations. They were kept when energies were below 10 kcal/mol, and superfluous conformations (RMSD > 0.75 Å) were removed. 5000 simulation cycles were applied with 5000 Large-scale Low Mode (LLMOD) search steps.

### 4.9. Circular Dichroism (CD)

CD spectra were recorded using a Jasco 810 UV-Vis spectropolarimeter, equipped with a CD/Fluorescence 426S/426L Peltier temperature controller. Peptide samples were dissolved in MeOH, and spectra were recorded at 200 µM. One sample was dissolved in H_2_O with 5% TFE. The following parameters were used: sensitivity (standard, 100 mdeg); start (260 nm); end (190 nm); data pitch (0.5 nm); scanning mode (continuous); scanning speed (200 nm/min); response (1 s); band width (1.0 nm); and accumulation (3). A blank spectrum of the buffer was subtracted from all recordings, and molar ellipticity values were calculated from experimental ellipticity using the Equation (3):

(3) $$\theta =\;\frac{\theta_{\exp}\cdot 10^{6}\;}{b\;\times C\;\times n}$$

where θ is the molar ellipticity in deg·cm^2^·dmol ^−1^, θ_exp_ is the measured ellipticity in mdeg, b is the optical path length in mm, C is the peptide concentration in µM and n is the number of residues in the peptide. After unit conversion, the spectrum was smoothed using the Savitzky-Golay method (convolution width = 21) and taken to zero at the far-UV region (λ = 260 nm).

## Figures and Tables

**Table 1:** Minimum inhibitory concentration (MIC) in µg/mL of the native MccJ25 and the new compound against eight Gram-negative strains [a]. The sensitive strains to the native MccJ25 are highlighted in bold.

Strains[a]	Native MccJ25	New Compound
***Escherichia coli* MDR 39255**	0.5	>256
*Escherichia coli* MDR 208691	>128	>128
***Escherichia coli* MDR 246415**	00625	>128
*Escherichia coli* MDR 239910	>128	>128
*Salmonella enterica* ATCC 14028	>128	>128
***Salmonella enterica* ATCC 13076**	<0.0625	>128
***Salmonella enterica* ATCC 49214**	0.8	>128
*Salmonella typhimurium* SY5015	>128	>128

**Table 2:** 1H chemical shift of the amide protons of the new compound that underwent peak overlapping [a]. From the total correlated spectroscopy (TOCSY) spectrum in methanol-d_3_ at 298 K.

Residue	δ (ppm)[a]	Residue	δ (ppm)[a]
Ser18	7.80	Glu8	7.90
Gly4	7.80	Ile13	7.89
Tyr9	7.84	Ala3	8.44
His5	7.79	Val11	8.42
		Gly12	8.39

## Supplementary Materials

Supplementary Materials can be found at https://www.mdpi.com/1422-0067/20/20/5152/s1.
