# Supplementary material for: Chemical Modification of Microcin J25 Reveals New Insights on the Stereospecific Requirements for Antimicrobial Activity
Source: Int J Mol Sci. 2019 Oct 17;20(20):5152. doi: 10.3390/ijms20205152 (PMC6829517; doi:10.3390/ijms20205152)
Supplement: Supplementary file 1 [file ijms-20-05152-s001.pdf]

Supplementary Material

Content

Materials and Methods 2

Characterization of Peptides 3

Native MccJ25 3

New Compound 3

Figures and Tables 3

References 25

Materials and Methods

Materials and reagents. Methanol was purchased from Scharlau, while acetonitrile was obtained from SDS. NaOH, 2,2,2-trifluoroethanol, pepsin, thermolysin were obtained from Sigma-Aldrich, while carboxypeptidase Y was purchased from Alfa Aesar. *E. coli* BL21 (DE3), which was used for heterologous expression, was purchased from Invitrogen. Bacterial strains were isolated from clinical specimens in the Microbiology Service of the Hospital Universitari de Bellvitge (Spain). Collistin sulfate was supplied by Zhejiang Shenghua Biok Biology Co. LTD, China. Mueller-Hinton Broth (MHB) was obtained from Scharlau (Spain).

Chemical Treatment. 0.1 mg of pure native MccJ25 was dissolved in 30 μL H_2O-ACN (1:1) and 10 μL of 0.5 M NaOH was added. The reaction mixture was stirred at 40ºC, and after 8, 16, 24 and 48 h an aliquot of sample was neutralized with 5 μL of 0.5 M HCl and analyzed by RP-HPLC and LC-MS. The optimum time for the treatment was found to be 16 h. For the isolation and purification of the new compound, 5.3 mg (2.5 μmol, 1 eq.) of pure native MccJ25 was dissolved in 400 μL of H_2O-ACN (1:1). Then, 30 μL of NaOH 0.5 M (pH 12) was added, and the mixture was stirred overnight at 40ºC. The reaction was then neutralized by adding 10 μL of HCl 0.5 M and purified by semi-analytical RP-HPLC using a Sunfire® C18 OBD™ Prep Column 5-μm (10×150 mm) reversed-phase column and a linear gradient (30% to 35% over 15 min) of ACN with a flow rate of 2.0 mL/min to obtain 1.50 mg of the new compound and 1.24 mg of native MccJ25 (which did not react) both with a purity > 99.9% (Fig. S10,11).

Stability Assays

Carboxypeptidase Y. Purified peptide (0.5 μg) was dissolved in 25 μL of MES buffer (50 mM MES, 1 mM $CaCl_2$ at pH 6.75). 10 μL of carboxypeptidase Y solution in MES buffer (1 mg/mL) was added to the peptide solution and incubated for 4 h at 25ºC. The reaction was quenched by the addition of 75 μL of ACN and then filtered and analyzed by LC-MS.

Pepsin. Purified peptide (20 μg) was dissolved in 150 μL HCl 0.01 M (pH 2). 40 μL of pepsin solution in HCl 0.01 M (1 mg/mL) was added to the peptide solution and incubated for 2-20 h at 37 ºC. The reaction was quenched by the addition of 0.05 M NaOH until pH 8-8.5, filtered and analyzed via LC-MS.

Thermolysin. Purified peptide (5-10 μg) was dissolved in 50 μL of buffer (50 mM Tris, 0.5 mM $CaCl_2$ at pH 8). 15 μL of thermolysin solution in buffer (1 mg/mL) was added to the peptide

solution and incubated for 30 min and 2 h at 70 ºC. The reaction was quenched by the addition of 4 M HCl until pH 2 was reached, and then filtered and analyzed by LC-MS.

Thermal. Purified peptide (10 μg) was dissolved in 100 μL of H_2O-ACN (1:1, v/v) and incubated at 95 ºC for 1, 2, 3 and 4 h. Samples were cooled to 4ºC and analyzed by LC-MS.

Characterization of Peptides

Native MccJ25

Analytical HPLC (linear gradient from 25% to 40% B in 40 min; t_R = 20.894, 99.9% purity) and LC-MS (ESI$^+$) *m/z* calculated for $C_{101}H_{139}N_{23}O_{27}$ $[M+H]^+$ 2106.02, found 2107.93. Production yield: 15.6 mg/L (Figure S19).

New Compound

Analytical HPLC (linear gradient from 25% to 40% B in 40 min; t_R = 18.791, 99.9% purity) and LC-MS (ESI$^+$) *m/z* calculated for $C_{101}H_{139}N_{23}O_{27}$ $[M+H]^+$ 2106.02, found 2107.78 (Figure S20).

Figures and Tables

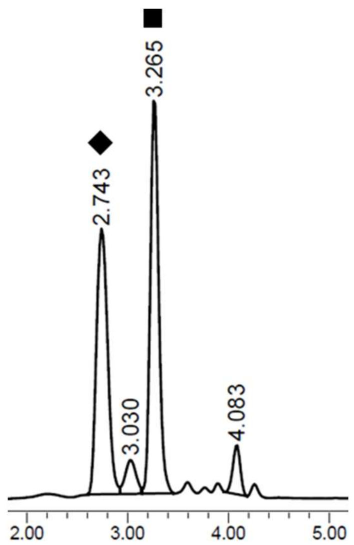

Figure S1. RP-HPLC chromatogram after the treatment with base of the native MccJ25. Conditions: XBridge C18 2.5 μm (4.6 mm x 75 mm) reversed-phase analytical column; linear gradient from 25% to 50% of ACN over 8 min at 25ºC. ■ = starting material, ◆ = new compound.

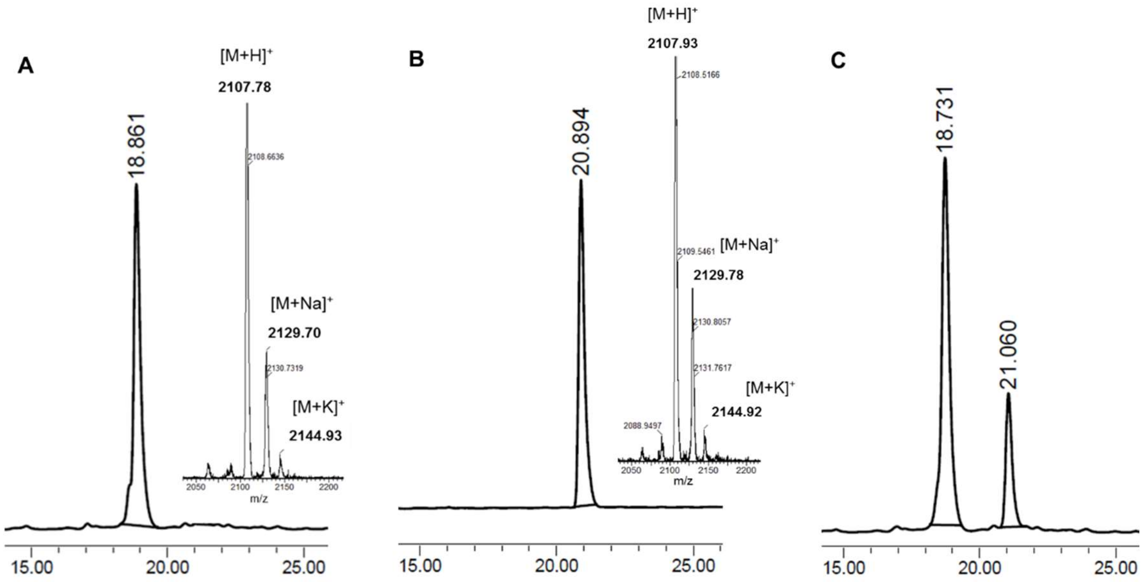

Figure S2. RP-HPLC chromatograms and ESI+ spectra of: (A) the new compound after treatment with a base, (B) native MccJ25 and (C) co-injection of both. Conditions: Phenomenex Luna C18 5 μm (4.6 mm x 250 mm) reversed-phase analytical column; linear gradient from 25% to 40% of ACN over 40 min at 60ºC.

Strains	FICi
***E. coli* MDR 39255**	0.40
***S. enterica* ATCC 13076**	0.41
***E. coli* MDR 208691**	0.52
***E. coli* MDR 239910**	0.54

Table S1. Fractional Inhibitory Concentration index (FICi) values of the new compound in combination with colistin.

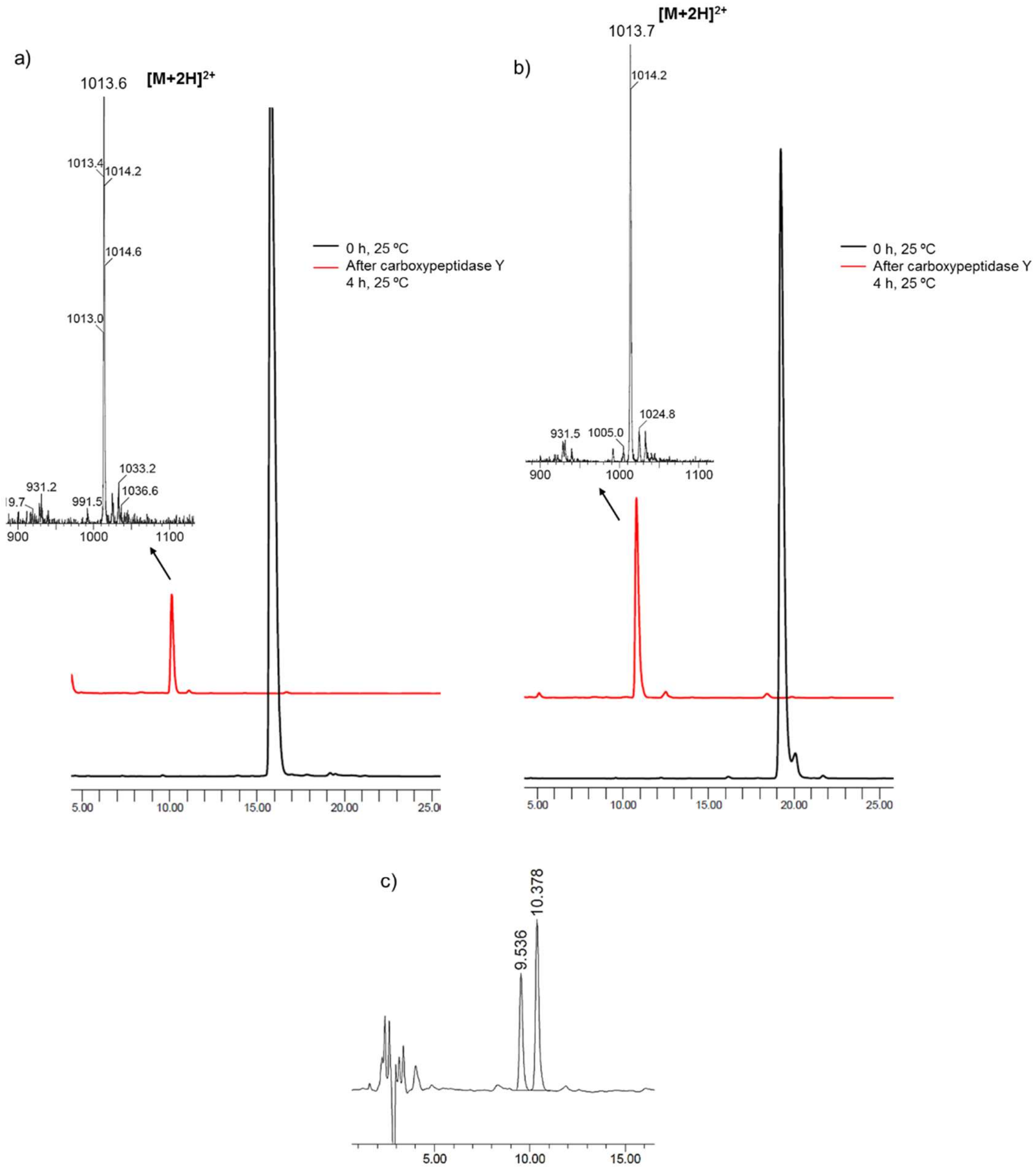

Figure S3. RP-HPLC chromatograms and ESI+ spectra of the carboxypeptidase Y assay of the a) new compound, b) native MccJ25 and c) co-injection of the two peptides after the carboxypeptidase Y cleavage. Conditions: Phenomenex Luna C18 5 µm (4.6 mm x 250 mm) reversed-phase analytical column; linear gradient from 25% to 40% of ACN over 40 min at 25ºC.

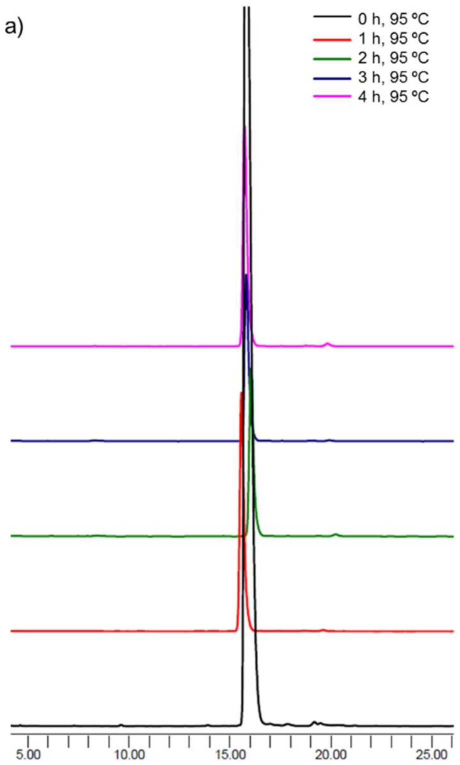

Figure S4. RP-HPLC chromatograms of the heat stability at 95ºC of the a) new compound and b) native MccJ25. Conditions: Phenomenex Luna C18 5 μm (4.6 mm x 250 mm) reversed-phase analytical column; linear gradient from 25% to 40% of ACN over 40 min at 25ºC.

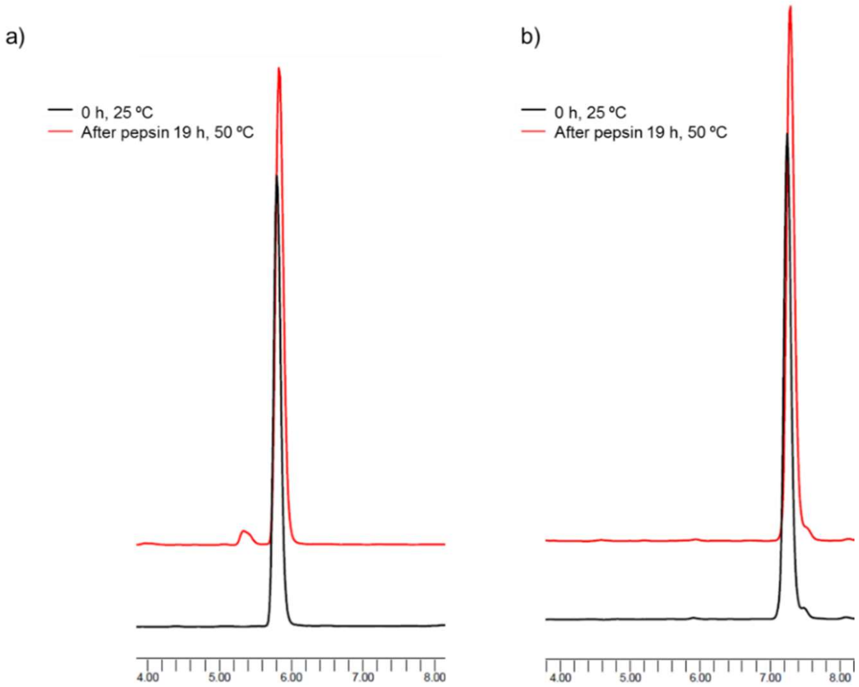

Figure S5. RP-HPLC chromatograms of the pepsin assay of the a) new compound and b) native MccJ25. Conditions: XBridge C18 2.5 μm (4.6 mm x 75 mm) reversed-phase analytical column; linear gradient from 25% to 35% of ACN over 8 min at 25ºC.

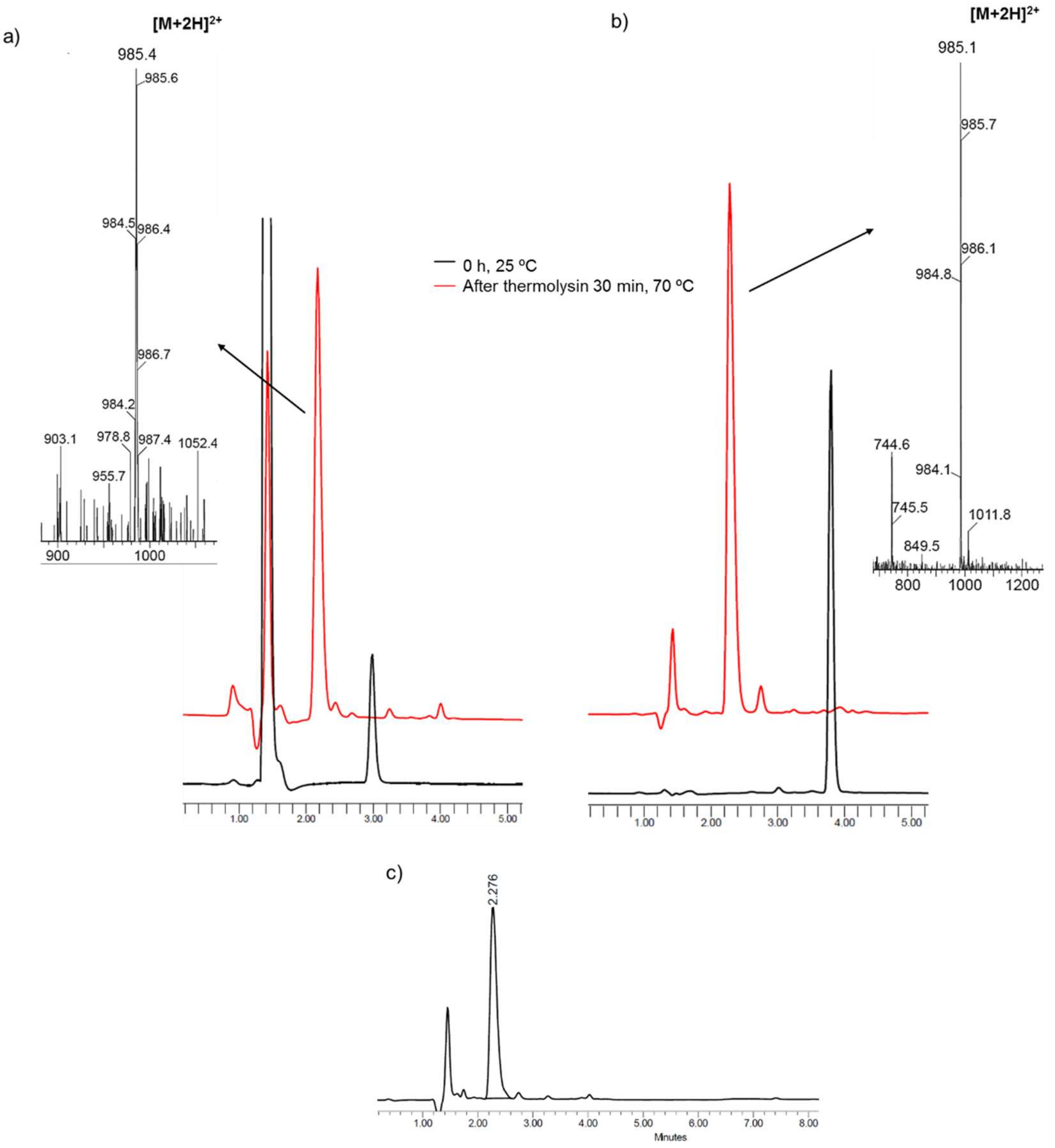

Figure S6. RP-HPLC chromatograms and ESI+ spectra after 30 min of thermolysin treatment at 70ºC in 50 mM TRIS pH 8, 0.5 mM $CaCl_2$ buffer. a) New compound, b) native MccJ25 and c) co-injection of the two peptides after the thermolysin cleavage. Conditions: XBridge C18 2.5 µm (4.6 mm x 75 mm) reversed-phase analytical column; linear gradient from 30% to 60% of ACN over 8 min at 25ºC.

a)

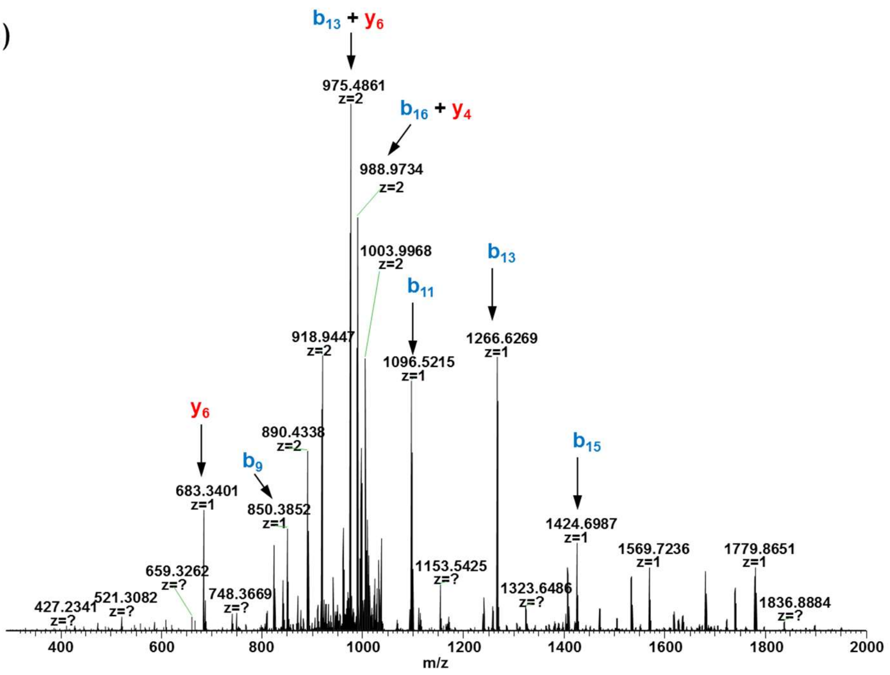
b13 + y6
975.4861
z=2
b16 + y4
988.9734
z=2
1003.9968
z=2
b13
b11
918.9447
z=2
1266.6269
z=1
1096.5215
z=1
y6
890.4338
z=2
b9
683.3401
z=1
850.3852
z=1
b15
1424.6987
z=1
1569.7236
z=1
1779.8651
z=1
659.3262
z=?
1153.5425
z=?
1323.6486
z=?
427.2341
z=?
521.3082
z=?
748.3669
z=?
1836.8884
z=?
400
600
800
1000
1200
1400
1600
1800
2000
m/z

b)

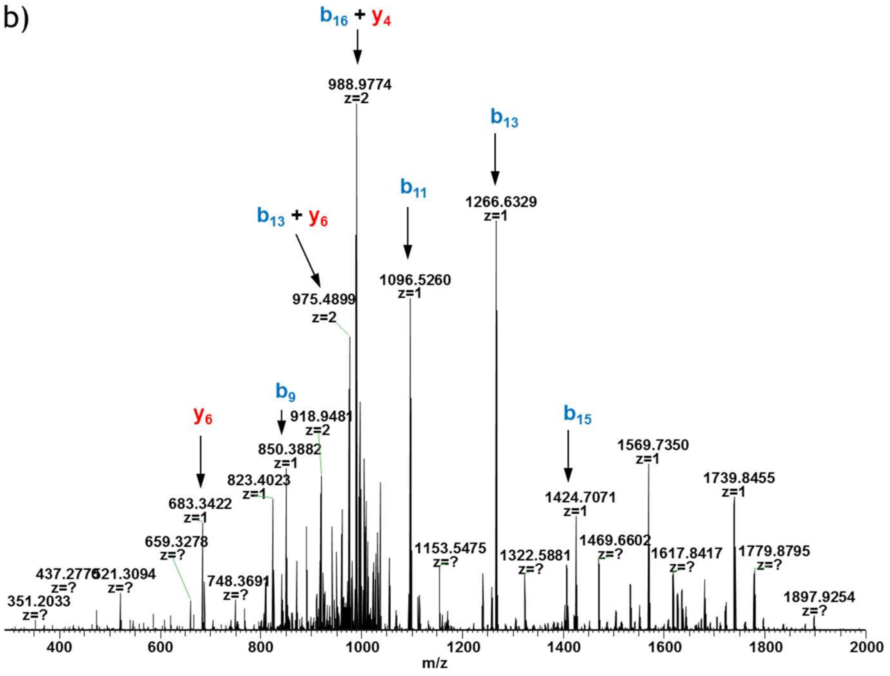
b16 + y4
988.9774
z=2
b13
b11
1266.6329
z=1
b13 + y6
1096.5260
z=1
975.4899
z=2
b9
y6
918.9481
z=2
b15
850.3882
z=1
1569.7350
z=1
823.4023
z=1
1739.8455
z=1
683.3422
z=1
1424.7071
z=1
659.3278
z=?
1469.6602
z=?
1153.5475
z=?
1322.5881
z=?
1617.8417
z=?
1779.8795
z=?
437.2776
z=?
521.3094
z=?
748.3691
z=?
351.2033
z=?
1897.9254
z=?
400
600
800
1000
1200
1400
1600
1800
2000
m/z

c)

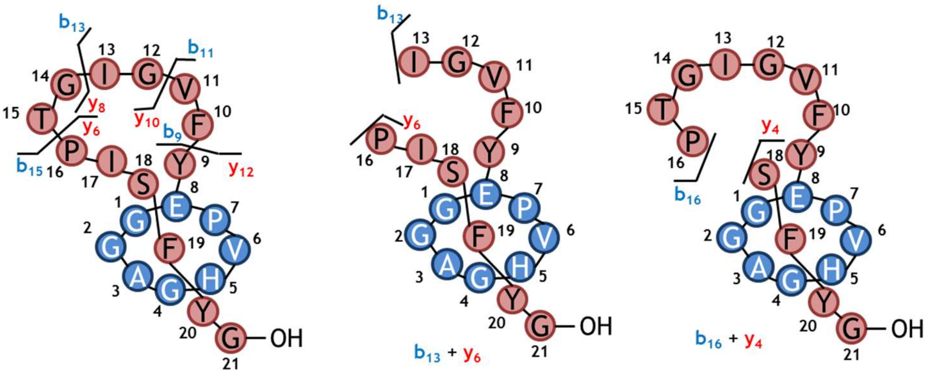

Figure S7. MS² fragmentation spectrum of the: a) new compound, b) native MccJ25 and c) schematic representation of the fragmentations derived from the MS² analysis.

Peptide	Most intense fragmentation	Description
New compound	$b_{13} + y_6$	Fragmentation around N-terminus Pro16. Loss of Gly14 and Thr15
Native MccJ25	$b_{16} + y_4$	Fragmentation around C-terminus of Pro16. Loss of Ile17

Table S2. Summary of the main MS² fragmentations

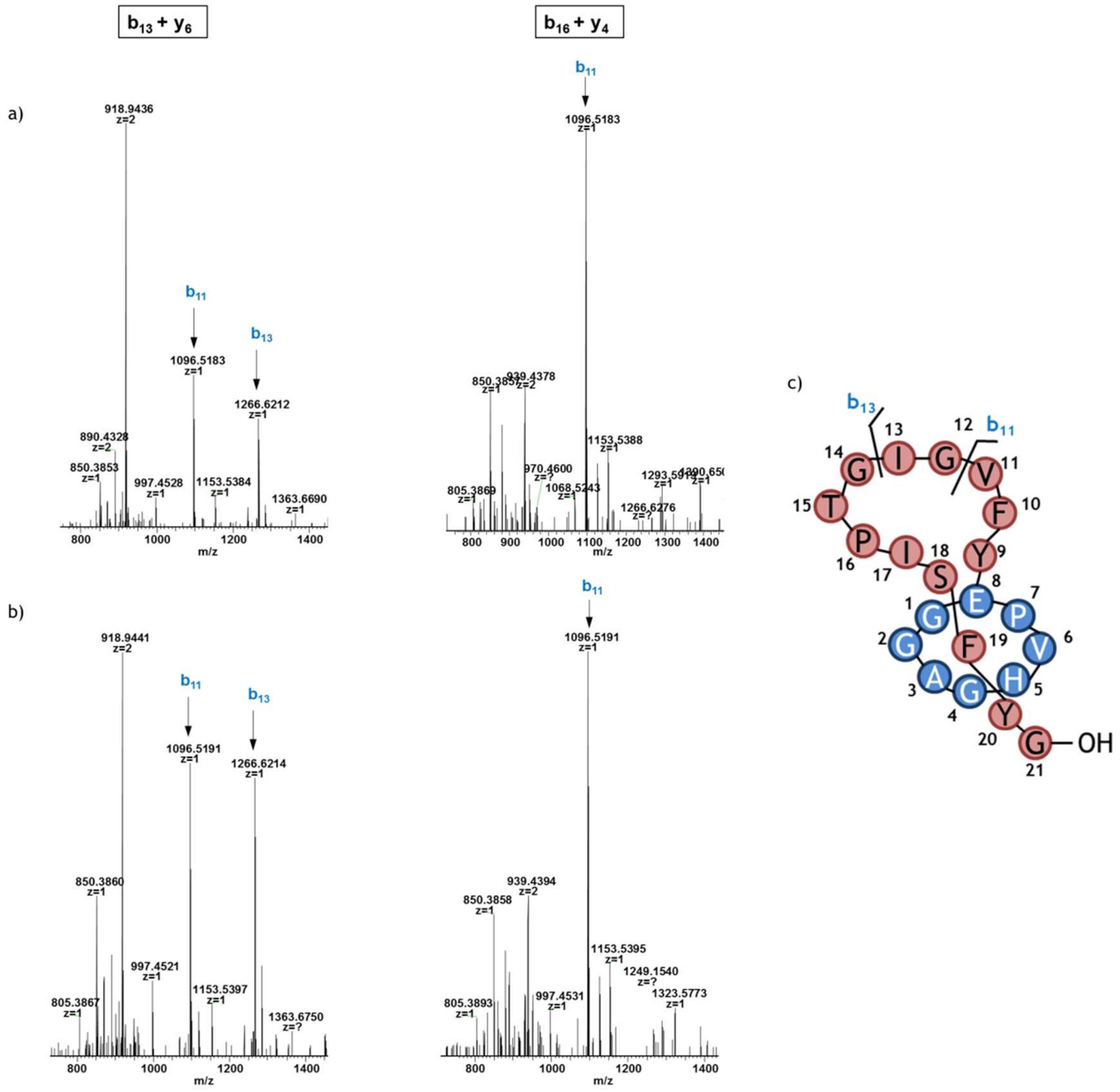

Figure S8. MS^3 spectrum of the $b_{13} + y_6$ and $b_{16} + y_4$ fragments of the: a) new compound, b) native MccJ25 and c) schematic representation of the fragmentations derived from the MS^3 analysis.

Peptides	Charge state	no. of conformations	CCS (Å^2) (Ratio (%))
New compound	$[M + 2H]^{2+}$	4	315 (11)/370 (40)/**396 (47)**/442
	$[M + 3H]^{3+}$	3	361 (10)/**382 (85)**/435 (5)
	$[M + 4H]^{4+}$	2	**375 (77)**/409 (23)
Native MccJ25	$[M + 2H]^{2+}$	4	315 (14)/360 (14)/**379 (67)**/437 (4)
	$[M + 3H]^{3+}$	3	363 (14)/**379 (75)**/427 (11)
	$[M + 4H]^{4+}$	2	**380 (77)**/417 (23)
Native MccJ25[1]	$[M + 2H]^{2+}$	3	**373 (63)**/383 (27)/393 (10)
	$[M + 3H]^{3+}$	1	383 (100)
	$[M + 4H]^{4+}$	2	**404 (88)**/442 (12)

Table S3. Number of conformations and CCS with 400 mM of sulfolane. Charge state $[M + H]^+$ is not shown because is a very unfolded structure with the highest drift time values and is not relevant for the comparison. Each CCS was calculated based on the drift times.[2] When several conformations were evidenced, the major species is highlighted in bold. Reported CCS values of native MccJ25 from previous work.[1]

	$[M + 2H]^{2+}$	$[M + 3H]^{3+}$	$[M + 4H]^{4+}$		
**Peptides**	**CCS (Ω, Å^2)**	**CCS (Ω, Å^2)**	**CCS (Ω, Å^2)**	**ΔΩ (Å^2)**	**ΔΩ/Ω (%)**
New compound	396	382	375	21	5.3
Native MccJ25	379	379	380	1	0.3
Native MccJ25[1]	373	383	404	31	7.7

Table S4. CCS values (Ω) of the main conformation from the ion mobility spectra. Range of CCS (ΔΩ) and ΔΩ/Ω parameter that indicates the relative range of CCS covered by all protonated molecule. Reported CCS values of native MccJ25 from previous work.[1]

Residue	Atom	δH	δC	Residue	Atom	δH	δC
Gly1	NH	7.93		Gly12	NH	8.16	
	α	4.31; 3.54	43.82		α	3.99; 3.76	43.47
Gly2	NH	8.98		Ile13	NH	8.32	
	α	4.22; 3.87	45.24		α	4.16	59.37
Ala3	NH	8.52			β	1.93	36.31
	α	4.67	49.06		γ	1.20; 1.54	25.70
	β	1.31	18.05		γ	0.93	15.66
Gly4	NH	7.74			δ	0.89	10.94
	α	4.05; 3.52	43.71	Gly14	NH	8.44	
His5	NH	7.50			α	4.06; 3.62	43.95
	α	4.66	54.11	Thr15	NH	7.90	
	β	3.27; 2.92	29.56		α	4.73	58.04
	δ	7.33	118.13		β	4.10	68.27
	ε	8.67	134.57		γ	1.21	19.59
Val6	NH	8.75		Pro16	α	4.49	61.02
	α	4.70	57.83		β	1.83; 1.72	30.57
	β	1.79	31.86		γ	2.16; 1.96	25.54
	γ	1.10	19.42		δ	4.01; 3.86	48.79
	γ	0.85	19.08	Ile17	NH	7.97	
Pro7	α	4.22	61.94		α	4.44	58.41
	β	1.80; 1.69	30.89		β	1.87	34.81
	γ	2.26; 1.99	26.36		γ	1.08; 1.43	25.72
	δ	4.13; 3.81	49.06		γ	0.85	16.16
Glu8	NH	8.34			δ	0.85	11.70
	α	4.40	53.67	Ser18	NH	7.35	
	β	1.68; 1.68	27.95		α	4.39	56.59
	γ	1.96; 1.84	33.24		β	4.07; 3.85	62.00
Tyr9	NH	7.33		Phe19	NH	8.86	
	α	4.56	55.69		α	5.43	56.79
	β	2.88; 2.65	38.76		β	2.57; 2.57	42.97
	δ	6.94	131.09		δ	6.87	130.19
	ε	6.59	115.77		ε	7.07	128.62
Phe10	NH	8.29			ζ	7.17	129.01
	α	4.86	55.62	Tyr20	NH	9.50	
	β	2.94; 2.76	39.86		α	4.89	55.47
	δ	7.07	130.02		β	2.99; 2.99	39.12
	ε	7.13	127.38		δ	6.95	131.45
	ζ	7.16	127.44		ε	6.69	116.03
Val11	NH	8.26		Gly21	NH	8.58	
	α	4.28	59.54		α	3.81; 3.73	43.01
	β	2.13	32.03				
	γ	0.92	19.62				
	γ	0.91	18.22				

Table S5. Chemical shifts of ^{1}H and ^{13}C (ppm) for the native MccJ25 in CD_3OH at 298K.

Residue	Atom	δH	δC	Residue	Atom	δH	δC
**Gly1**	NH	7.97		**Gly12**	NH	8.39	
	α	4.21, 3.64	43.76		α	4.22, 3.57	43.70
**Gly2**	NH	8.86		**Ile13**	NH	7.89	
	α	4.33, 3.82	45.09		α	4.29	59.68
**Ala3**	NH	8.43			β	1.92	37.52
	α	4.67	49.01		γ	1.48, 1.16	25.66
	β	1.31	18.04		γ	0.95	15.83
**Gly4**	NH	7.80			δ	0.80	10.76
	α	4.09, 3.58	43.74	**Gly14**	NH	8.57	
**His5**	NH	7.79			α	3.73, 4.07	43.77
	α	4.48	55.71	**Thr15**	NH	7.59	
	β	3.31, 2.87	29.57		α	4.63	57.96
	δ	7.29	117.54		β	4.03	68.39
	ε	8.51	133.98		γ	1.21	19.88
**Val6**	NH	8.95		**Pro16**	α	4.67	61.07
	α	4.77	57.52		β	1.79, 1.82	30.27
	β	1.91	32.15		γ	2.10, 1.91	25.48
	γ	1.15	19.48		δ	3.91, 3.82	48.78
	γ	0.87	18.83	**Ile17**	NH	8.08	
**Pro7**	α	4.33	61.86		α	4.34	58.13
	β	1.84, 1.53	30.40		β	1.77	37.82
	γ	2.21, 1.95	26.63		γ	1.35, 1.05	25.53
	δ	4.04, 3.76	49.04		γ	0.77	15.55
**Glu8**	NH	7.90			δ	0.75	11.35
	α	4.28	53.45	**Ser18**	NH	7.80	
	β	1.78	27.69		α	4.36	57.50
	γ	2.01, 1.93	34.27		β	3.24, 3.82	62.90
**Tyr9**	NH	7.84		**Phe19**	NH	8.84	
	α	4.51	55.89		α	5.52	55.23
	β	2.84, 2.60	38.22		β	2.68, 2.54	49.85
	δ	6.95	131.37		δ	6.94	130.48
	ε	6.60	115.86		ε	7.11	127.07
**Phe10**	NH	8.31			ζ	7.11	128.47
	α	4.64	55.77	**Tyr20**	NH	9.55	
	β	3.09, 2.94	38.54		α	4.97	55.61
	δ	7.20	129.77		β	3.03	39.43
	ε	7.13	127.32		δ	6.97	131.35
	ζ	7.24	129.19		ε	6.74	116.18
**Val11**	NH	8.42		**Gly21**	NH	8.49	
	α	3.87	61.74		α	3.73, 3.49	44.54
	β	2.06	30.71				
	γ	0.89	19.18				
	γ	0.89	19.18				

Table S6. Chemical shifts of ^{1}H and ^{13}C (ppm) for the new compound in CD_3OH at 298K.

Residue	$^3J_{NH\text{-}\alpha H}$ (Hz)	Φ	Residue	$^3J_{NH\text{-}\alpha H}$ (Hz)	Φ
**Gly1**	8.2; 3.9	-150.5	**Gly12**	ND	
**Gly2**	6.6	-137.7	**Ile13**	8.4	-151.6
**Ala3**	8.5	-152.9	**Gly14**	6.2	-135.2
**Gly4**	7.3	-143.1	**Thr15**	8.3	-151.6
**His5**	10.0	-171.9	**Pro16**		
**Val6**	9.1	-158.4	**Ile17**	9.5	-163.7
**Pro7**			**Ser18**	*7.2*	*-142.2*
**Glu8**	9.1	-158.4	**Phe19**	10.9	--
**Tyr9**	*7.2*	*-142.2*	**Try20**	7.9	-148.2
**Phe10**	8.9	-156.9	**Gly21**	5.8	-132.1
**Val11**	8.5	-152.9			

Table S7: Coupling constant $^3J_{NH\text{-}\alpha H}$ and dihedral torsion angles (◎) from the 1D spectrum for the native MccJ25 in CD_3OH at 298K. Values in italics are approximated due to peak overlapping. ND = not determined.

Residue	$^3J_{NH-\alpha H}$ (Hz)	Φ	Residue	$^3J_{NH-\alpha H}$ (Hz)	Φ
**Gly1**	6.2	-135.2	**Gly12**	6.2	-135.2
**Gly2**	*8.5*	*-152.9*	**Ile13**	*7.2*	*-142.2*
**Ala3**	*8.7*	*-155.5*	**Gly14**	6.1	-134.4
**Gly4**	ND		**Thr15**	7.1	-141.3
**His5**	ND		**Pro16**		
**Val6**	9.1	-158.4	**Ile17**	9.3	-161.8
**Pro7**			**Ser18**	ND	
**Glu8**	*7.2*	*-142.2*	**Phe19**	*8.5*	*-152.9*
**Tyr9**	7.5	-145.1	**Try20**	7.8	-147.1
**Phe10**	7.0	-141.3	**Gly21**	5.8	-132.1
**Val11**	*8.7*	*-155.5*			

Table S8: Coupling constant $^3J_{NH-\alpha H}$ and dihedral torsion angles (◎) from the 1D spectrum for the new compound in CD_3OH at 298K. Values in italics are approximated due to peak overlapping. ND = not determined.

β-hairpin	NOEs	Native MccJ25	New compound
**Val6 - Pro7** **Tyr20 - Phe19**	αH Pro7 – αH Phe19	X	X
	αH Pro7 - βH Phe19	X	X
	αH Phe19 – αH Pro7	X	X
	δH Pro7 – αH Val6	X	X
**Phe10 - Val11** **Pro16 - Thr15**	αH Pro16 – αH Phe10	X	-
	αH Val11 – δH Pro16	X	-
	δH Pro16 – αH Val11	X	-
	δH Pro16 – αH Thr15	X	X

Table S9. NOE cross-peaks of the two β-hairpin of the two peptides.

Val11	NOE	Native MccJ25	New compound
	αH Phe10	X	X
	**βH Phe10**	-	X
	**NH Phe10**	-	X
**NH**	**NH Ile17**	X	X
	δH Ile17	X	X
	γH Ile17	-	X
	**NH Thr15**	-	X
**α**	**δH Pro16**	X	-
	NH Gly12	X	X
**β**	**βH Pro16**	X	-
	**γH Pro16**	X	-
	**NH Gly12**	X	-
**γ**	βH Ile13	X	X
	αH Pro16	X	-
	**NH Gly12**	X	-
	αH Gly12	X	-
	βH Phe10	X	-
	δH Ile17	-	X
	εH Tyr9	-	X
	**αH Thr15**	-	X
	**βH Thr15**	-	X

Table S10. NOE cross-peaks for the residue Val11 for the native MccJ25 and the new compound. The most intense NOEs for the native MccJ25 are highlighted in black. The most remarkable cross-peaks observed for the new product are highlighted in red.

Val11	NOE	Native MccJ25	New compound
NH	αH Phe10	188133.1	798302.6
	βH Phe10	-	64860.0
	NH Phe10	-	243370.5
	NH Ile17	221695.1	w
α	NH Gly12	320263.2	547478.7

Table S11. Intensities of the main NOE cross-peaks of Val11 residue from the new compound. w = cross peak with very low intensity.

Atoms	Distance (Å)	Atoms	Distance (Å)
NH Val11 – βH Phe10	3.0	βH Tyr9 – βH Phe10	2.2
NH Val11 – NH Ile17	4.0	NH Phe10 – NH Tyr9	3.0
αH Val11 – NH Gly12	2.2	NH Phe10 – αH Tyr9	2.2
αH Val11 – βH Val11	2.2	NH Phe10 – βH Tyr9	2.2
αH Val11 – γH Val11	2.2	αH Phe10 – NH Val11	3.0
βH Val11 – βH Pro16	4.0	αH Ile13 – NH Tyr9	4.0
βH Val11 – γH Pro16	4.0	NH Gly12 – NH Ile13	2.2
γH Val11 – αH aThr15	4.0	NH Gly12 – NH Phe10	3.0
γH Val11- αH Val11	2.2	αH Gly12 – NH Ile13	2.2
γH Val11 – βH Thr15	4.0	NH Thr15 – NH Ile13	3.0
γH Val11 - δH Ile17	4.0	NH Thr15 – γH Ile13	3.0
NH His5 – NH Gly1	3.0	δH Pro16 – δH Tyr9	3.0
NH His5 – δH His5	3.0	δH Tyr20 – NH Gly21	2.2
αH Ser18 – NH Gly1	3.0	δH Phe19 – βH1 Ser18	3.0
αH Ser18 – NH Gly2	3.0	δH Phe19 – βH2 Ser18	2.2
NH Tyr9 – αH Glu8	2.2	βH Phe19 – δH Tyr9	3.0
NH Tyr9 – NH Ile17	3.0	αH Pro7 – αH Phe19	2.2
NH Tyr9 – NH Phe10	3.0	αH Pro7 – βH Phe19	2.2
αH Tyr9 – NH Phe10	2.2	δH Pro7 – αH Val6	2.2
αH Tyr9 – αH Phe10	2.2	αH His5 – γH Val6	4.0

Table S12. Constrained distances extracted from the NOESY spectrum of the new compound in CD_3OH at 298K. Force constant used of 100 kcal·mol^{-1}·Å^{-2}.

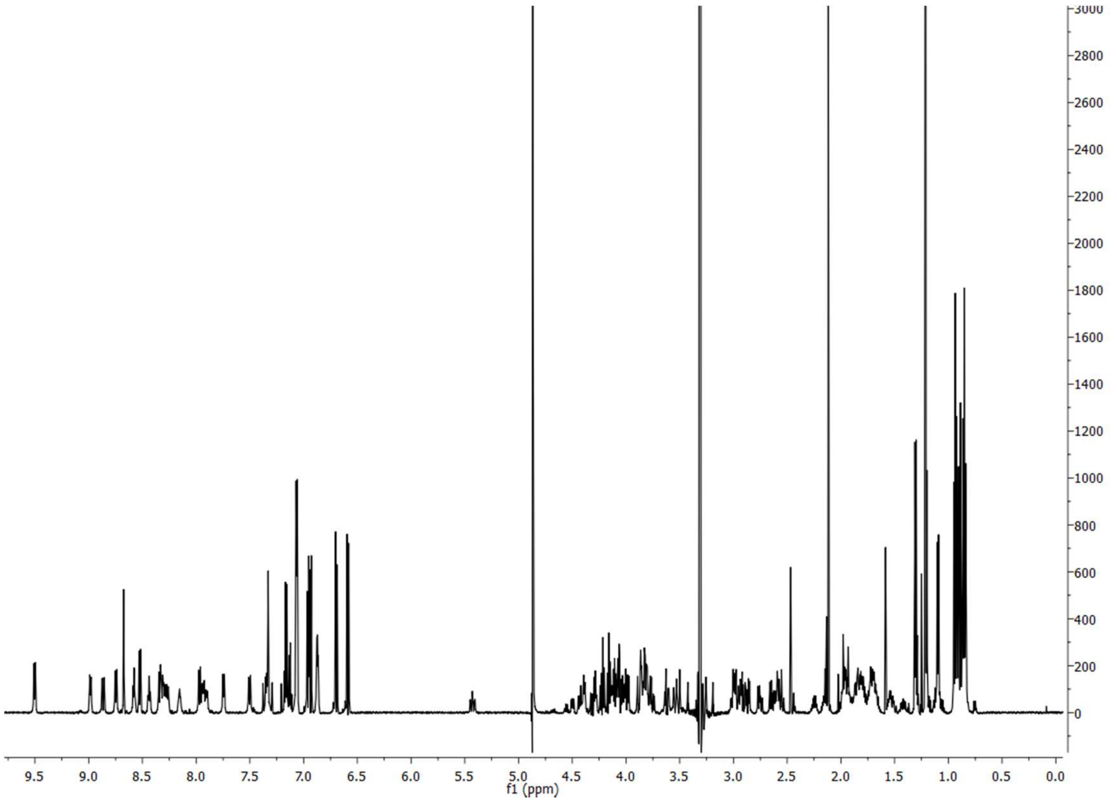

Figure S9. ^{1}H spectrum of the native MccJ25 in CD_3OH at 298K.

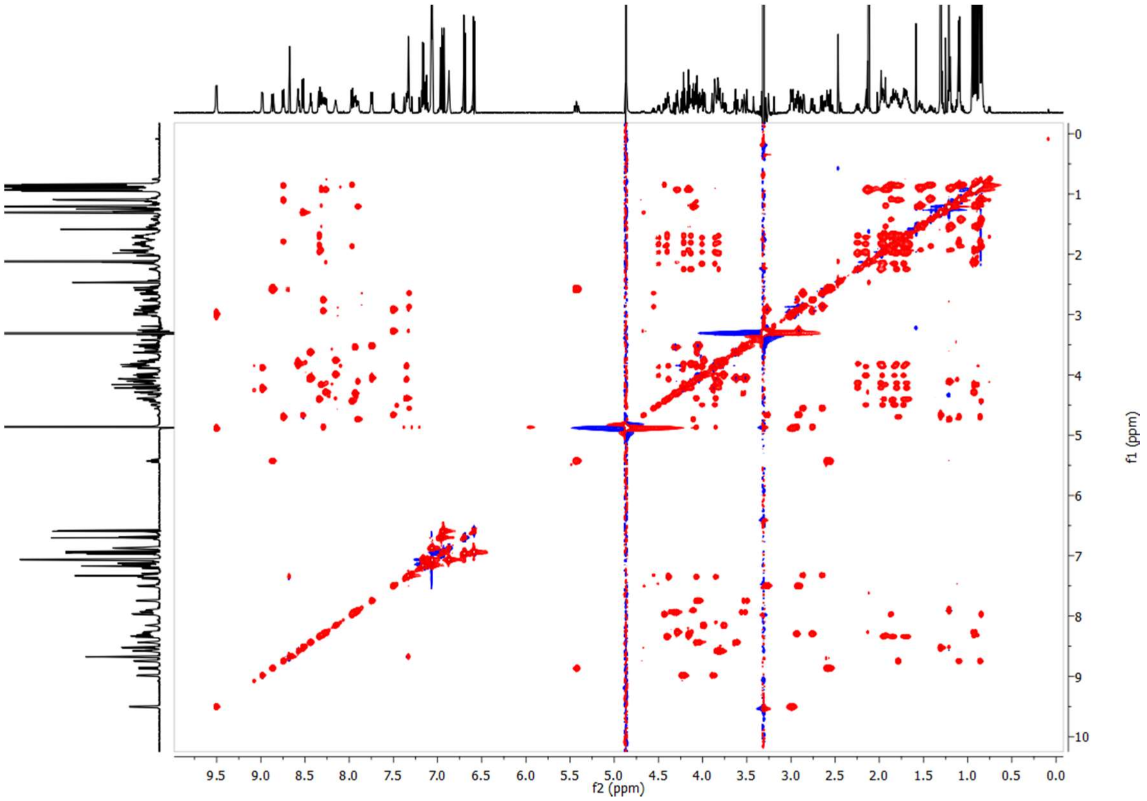

Figure S10. 2D TOCSY spectrum of the native MccJ25 in CD_3OH at 298K.

.

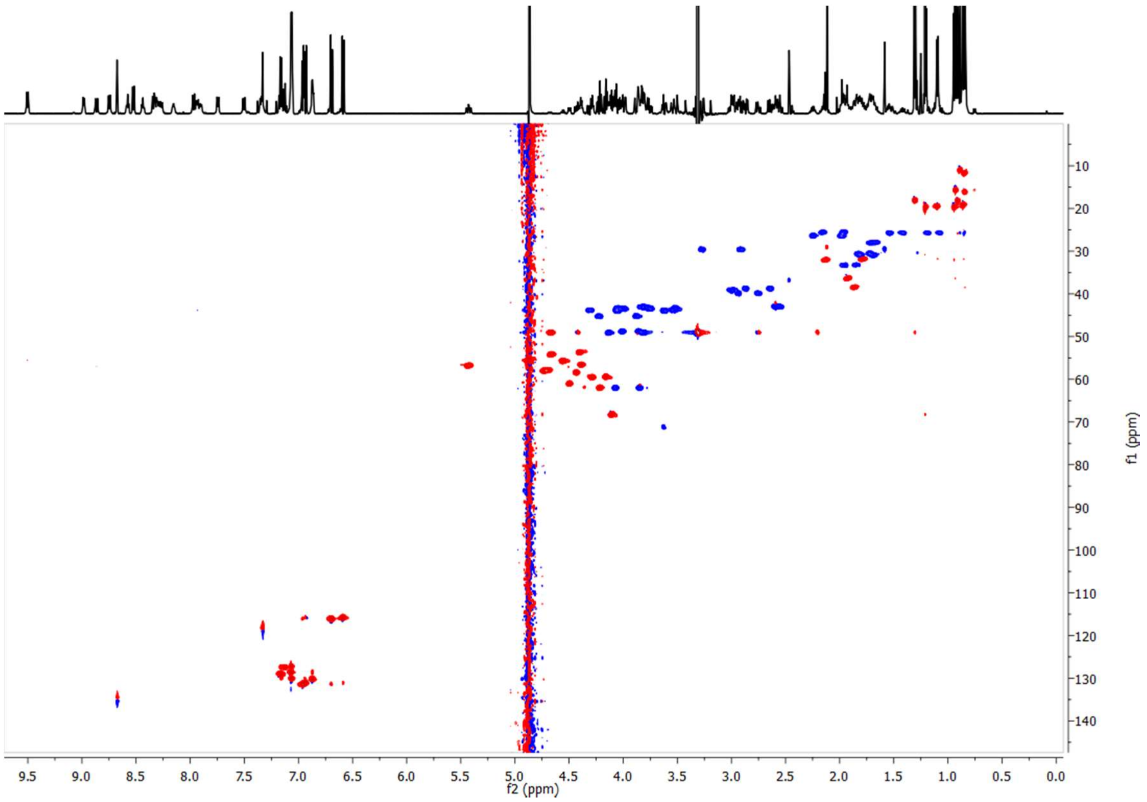

Figure S11. 2D ^1H-^{13}C HSQC spectrum of the native MccJ25 in CD_3OH at 298K.

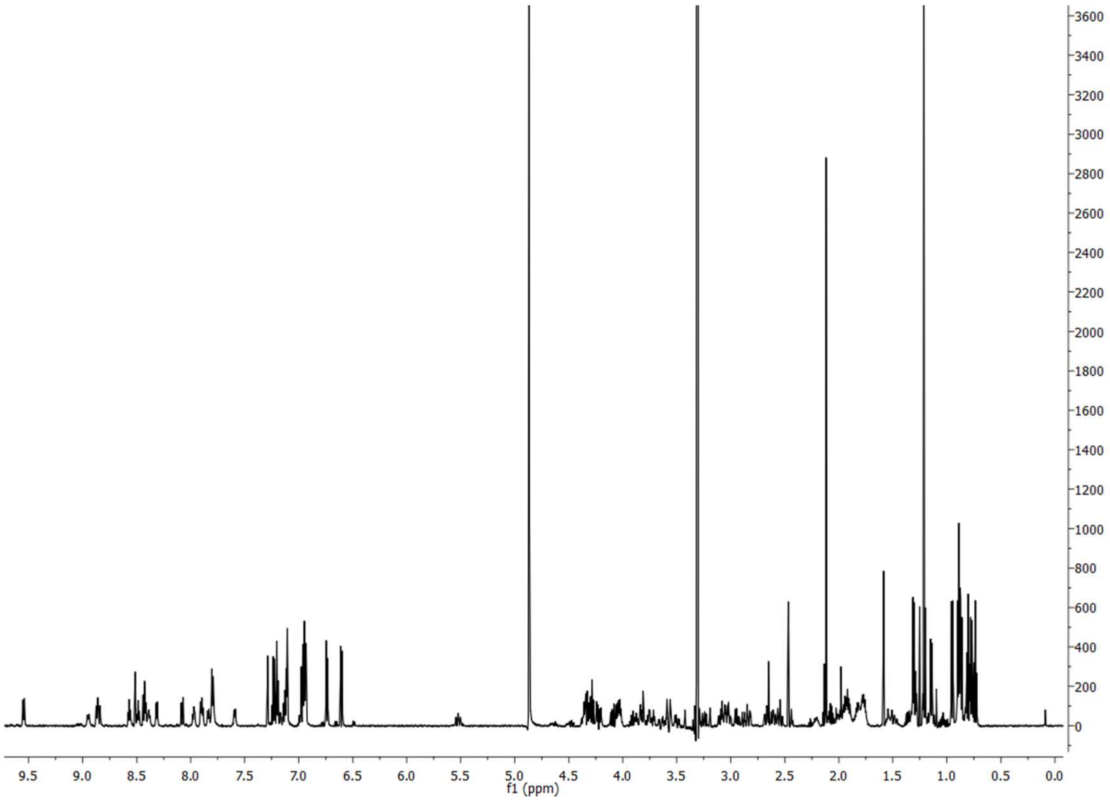

Figure S12. ^1H spectrum of the new compound in CD_3OH at 298K.

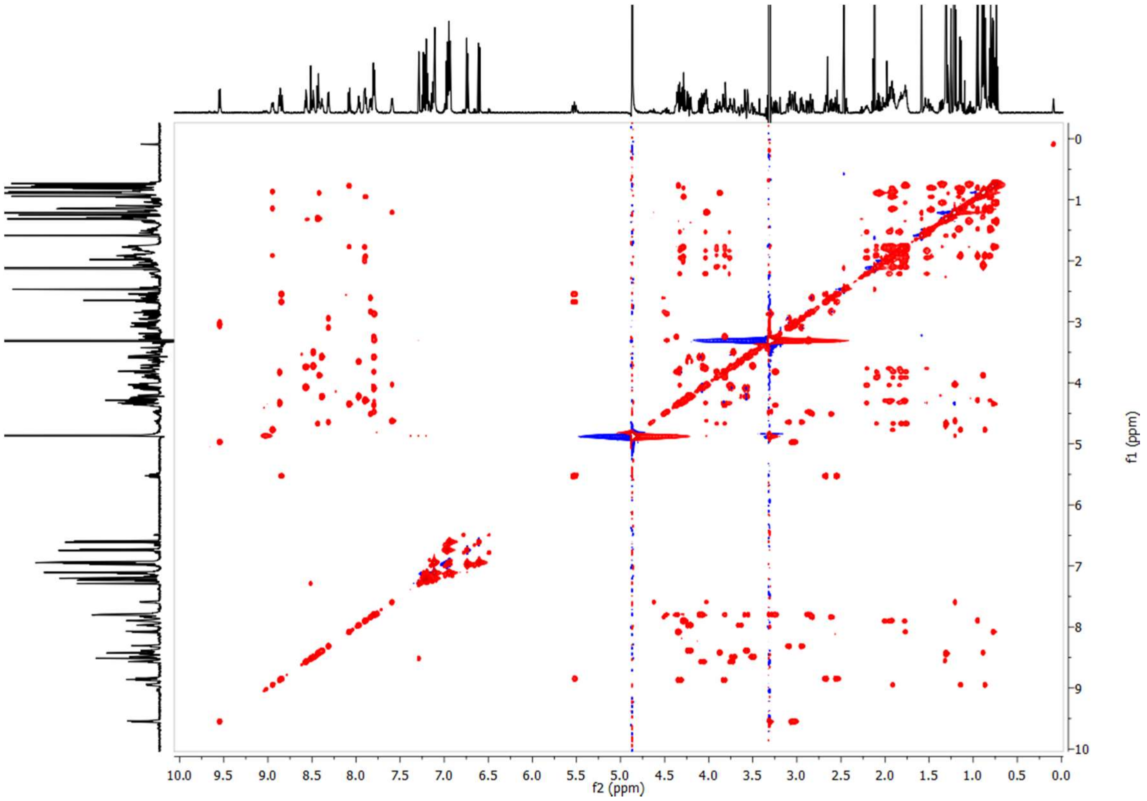

Figure S13. 2D TOCSY spectrum of the new compound in CD_3OH at 298K.

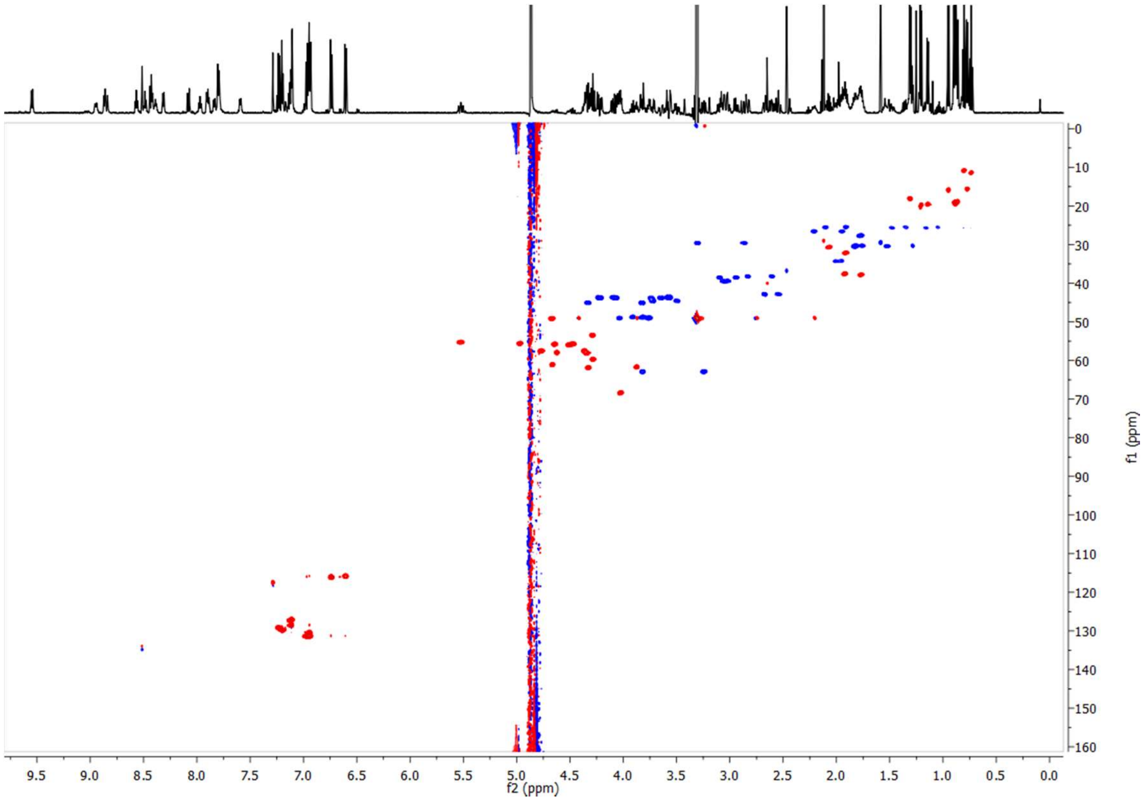

Figure S14. 2D ^1H-^{13}C HSQC spectrum of the new compound in CD_3OH at 298K.

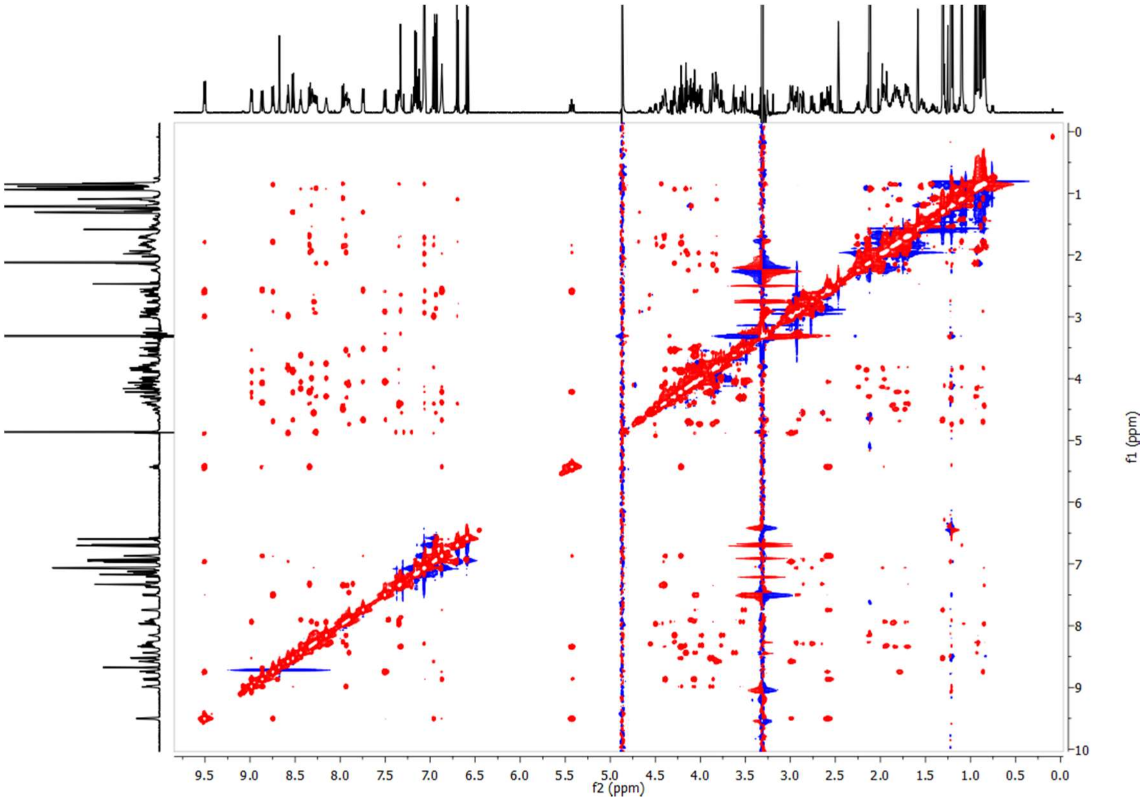

Figure S15. 2D NOESY spectrum of the native MccJ25 in CD_3OH at 298K.

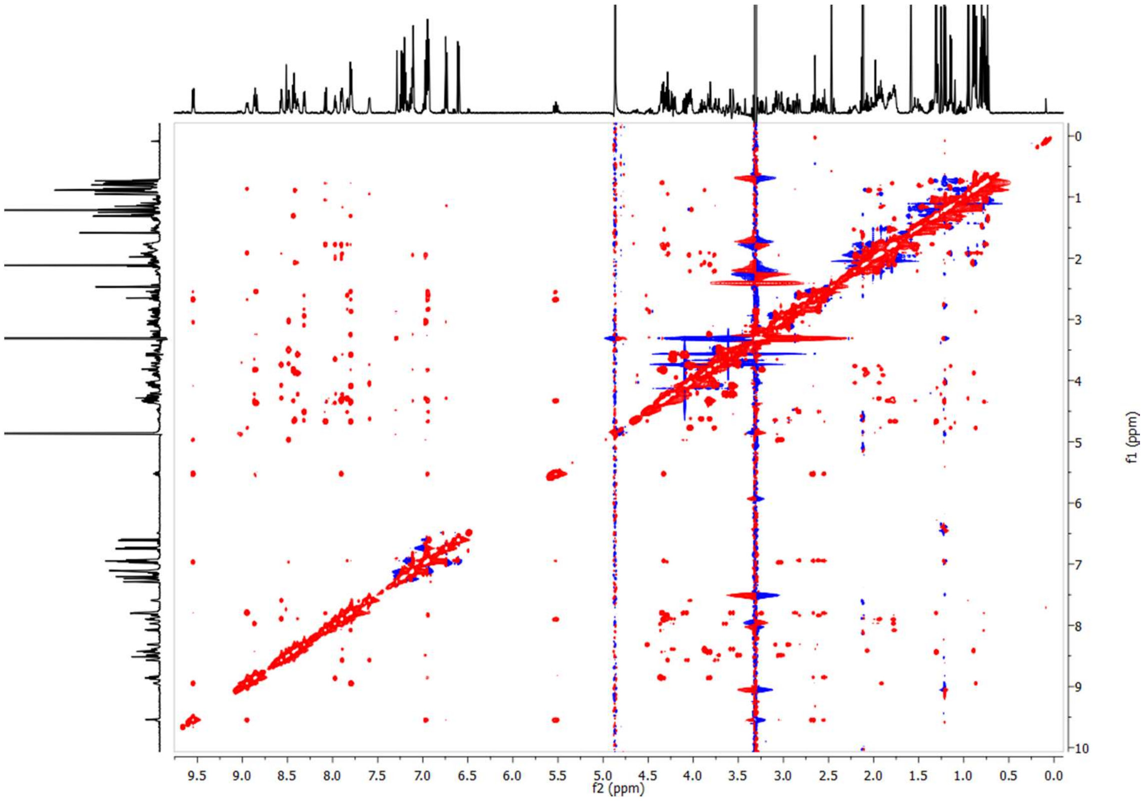

Figure S16. 2D NOESY spectrum of the new compound in CD_3OH at 298K.

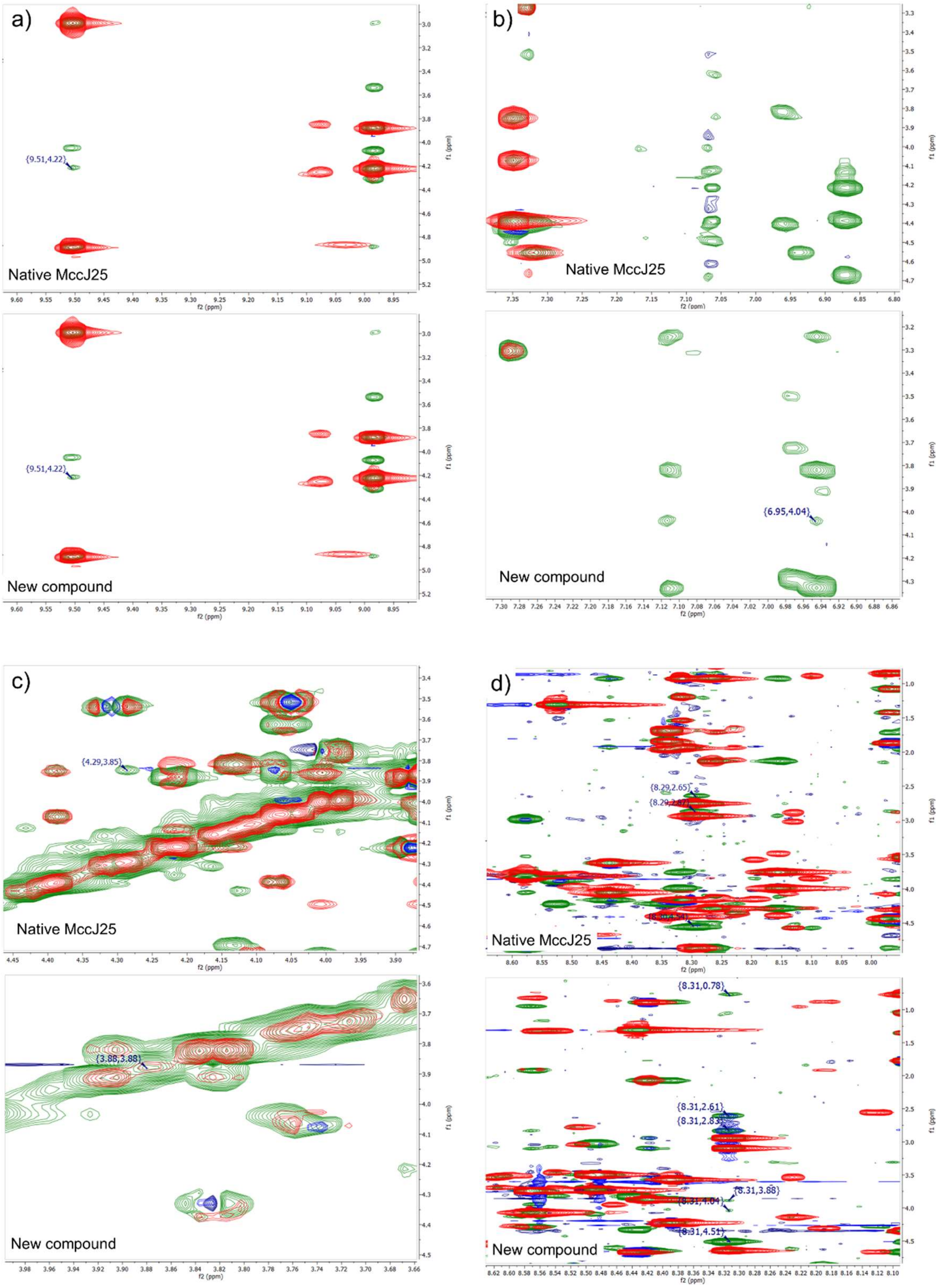

Figure S17. Superposition of expanded regions of 2D TOCSY (red) and NOESY (green) spectra of the native MccJ25 and new compound in MeOH-d_3. NOE cross-peaks are highlighted in the spectra: a) NH Tyr20 – αH Gly2, b) δH Tyr9 – αH Gly14, c) αH Val11 – δH Pro16 and d) NOEs of NH Phe10.

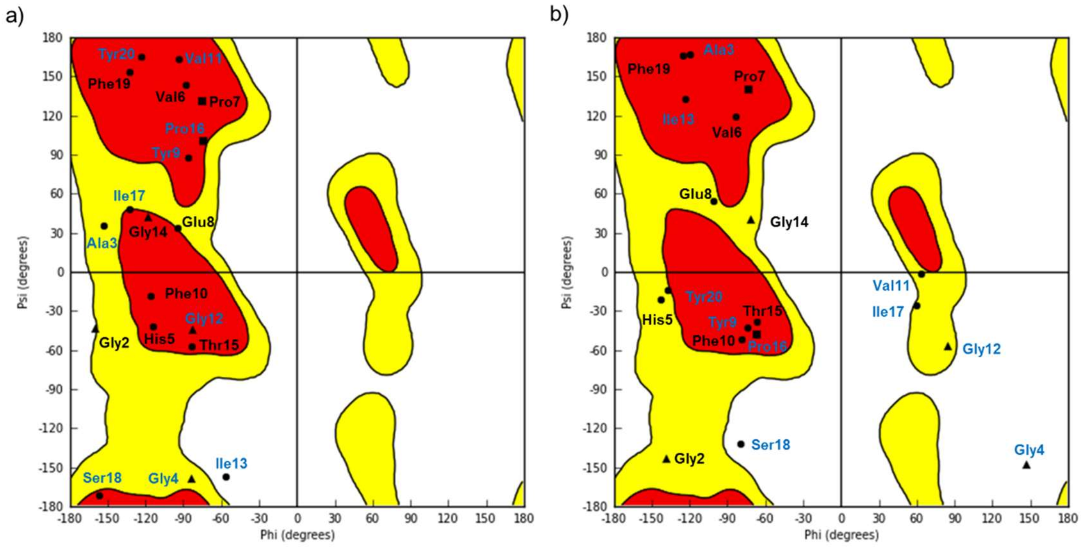

Figure S18. Ramachandran plot of the a) native MccJ25 (PDB code 1PP5) and b) lowest-energy structure derived from the NMR restraints of the new compound. The regions denoted in red are the most highly favored regions; in yellow are the additional allowed regions and in white the disallowed regions. The residues located in different region between both structures are shown in blue. The plot is generated using Maestro software.

Component	Amount
biotin	0.2 g
choline chloride	1.0 g
disodium adenosine 5′-triphosphate	0.3 g
folic acid	1.0 g
myo-inositol	2.0 g
nicotinamide	1.0 g
panthothenic acid	1.0 g
pyridoxal hydrochloride	1.0 g
riboflavin	0.1 g
thiamine	1.0 g
H_2O	300 mL

10 M NaOH were added to the resulting suspension until complete dissolution. The solution was then sterile filtered and stored at 4ºC

Table S13. M9 minimal medium Vitamin Mix composition

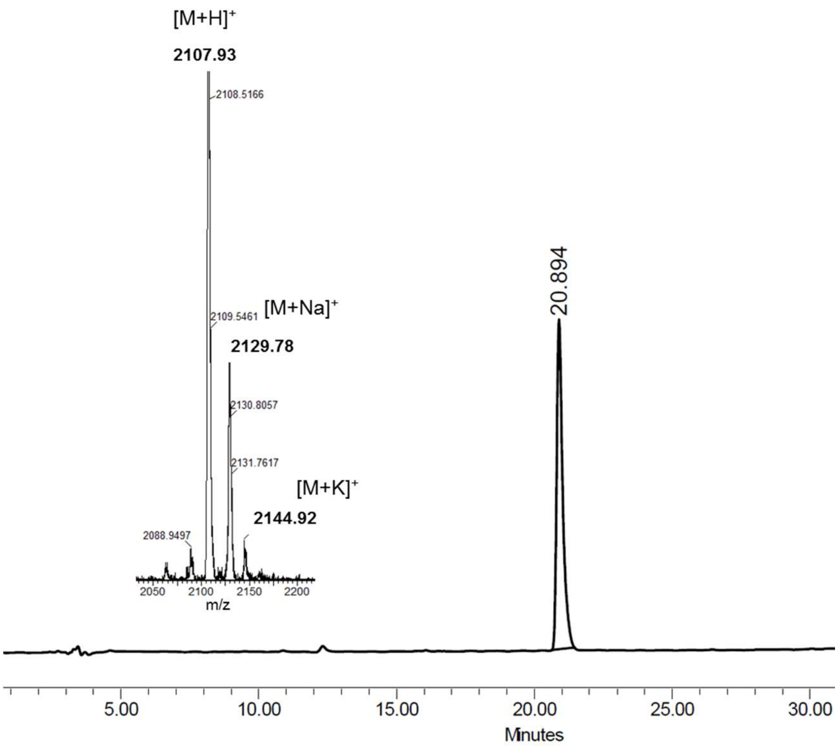

Figure S19. RP-HPLC chromatogram and MALDI-TOF spectrum of the native MccJ25. Conditions: Phenomenex Luna C18 5 µm (4.6 mm x 250 mm) reversed-phase analytical column; linear gradient from 25% to 40% of ACN over 40 min at 60ºC.

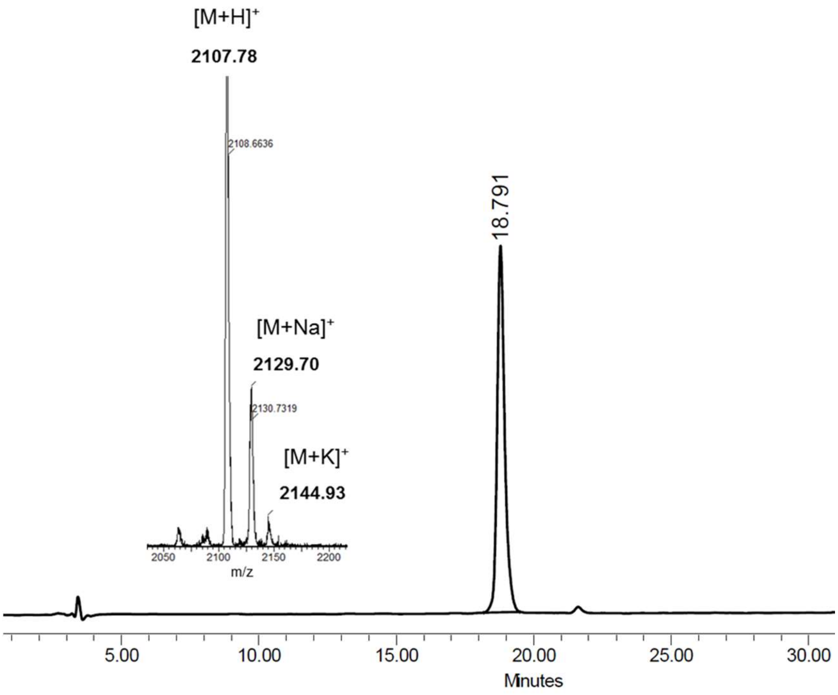

Figure S20. RP-HPLC chromatogram and MALDI-TOF spectrum of the new compound. Conditions: Phenomenex Luna C18 5 µm (4.6 mm x 250 mm) reversed-phase analytical column; linear gradient from 25% to 40% of ACN over 40 min at 60ºC.

References

1. Jeanne Dit Fouque, K.; Afonso, C.; Zirah, S.; Hegemann, J.D.; Zimmermann, M.; Marahiel, M.A.; Rebuffat, S.; Lavanant, H. Ion mobility-mass spectrometry of lasso peptides: Signature of a rotaxane topology. *Anal. Chem.* **2015**, *87*, 1166–1172.
2. Smith, D.; Knapman, T.; Campuzano, I.; Malham, R.; Berryman, J.; Radford, S.; Ashcroft, A. Deciphering drift time measurements from travelling wave ion mobility spectrometry-mass spectrometry studies. *Eur. J. Mass Spectrom.* **2009**, *15*, 113–130.
